# CENP-F stabilizes kinetochore-microtubule attachments and limits dynein stripping of corona cargoes

**DOI:** 10.1083/jcb.201905018

**Published:** 2020-03-24

**Authors:** Philip Auckland, Emanuele Roscioli, Helena Louise Elvidge Coker, Andrew D. McAinsh

**Affiliations:** 1 Centre for Mechanochemical Cell Biology & Division of Biomedical Sciences, Warwick Medical School, University of Warwick, Coventry, UK; 2 Computing and Advanced Microscopy Development Unit, Warwick Medical School, University of Warwick, Coventry, UK

## Abstract

Accurate chromosome segregation demands efficient capture of microtubules by kinetochores and their conversion to stable bioriented attachments that can congress and then segregate chromosomes. An early event is the shedding of the outermost fibrous corona layer of the kinetochore following microtubule attachment. Centromere protein F (CENP-F) is part of the corona, contains two microtubule-binding domains, and physically associates with dynein motor regulators. Here, we have combined CRISPR gene editing and engineered separation-of-function mutants to define how CENP-F contributes to kinetochore function. We show that the two microtubule-binding domains make distinct contributions to attachment stability and force transduction but are dispensable for chromosome congression. We further identify a specialized domain that functions to limit the dynein-mediated stripping of corona cargoes through a direct interaction with Nde1. This antagonistic activity is crucial for maintaining the required corona composition and ensuring efficient kinetochore biorientation.

## Teaser

A key step in accurate chromosome segregation is the removal of the outer corona of the kinetochore when microtubules are attached. Auckland et al. reveal how CENP-F stabilizes kinetochore-microtubule attachments and limits dynein-mediated corona stripping.

## Introduction

Accurate chromosome segregation during mitosis is dependent on a centromere-associated protein machine called the kinetochore. Kinetochores are built from the hierarchical assembly of two major complexes, the 16-subunit constitutive centromere-associated network, which makes multiple contacts with CENP-A nucleosomes and recruits the 10-subunit KNL1/Mis12/Ndc80 network (Musacchio and Desai, 2017). Multiple copies of these complexes give rise to the inner and outer kinetochore and are well established as mediators of microtubule attachment and spindle assembly checkpoint signaling (Monda and Cheeseman, 2018; Musacchio, 2015). Metazoan kinetochores also contain a region distal to the outer kinetochore called the fibrous corona, named for its appearance on electron micrographs (McEwen et al., 1993, 1998). The corona is a highly dynamic structure built from the Rod-Zw10-Zwlich (RZZ) complex, Spindly, centromere protein (CENP)-F, the molecular motors CENP-E and dynein, and the checkpoint proteins Mad1, Mad2, and Cyclin B (Allan et al., 2019; Maiato et al., 2004). At unattached kinetochores, the corona expands into a crescent-like structure that can even encircle the entire pair of sister chromatids (Hoffman et al., 2001; Magidson et al., 2015; Pereira et al., 2018; Sacristan et al., 2018; Thrower et al., 1996; Wynne and Funabiki, 2015; Wynne and Funabiki, 2016). This expansion is driven by a farnesylation-mediated conformational change in Spindly (Sacristan et al., 2018) and Mps1-dependent phosphorylation of Rod, both of which enable the self-assembly of RZZ-Spindly (RZZ-S) into a high-order meshwork (Pereira et al., 2018; Rodriguez-Rodriguez et al., 2018; Sacristan et al., 2018). The expanded corona is thought to provide a large surface area for the initial (lateral) capture of spindle microtubules by CENP-E and dynein motors (Sacristan et al., 2018). As microtubules form end-on kinetochore attachments, the corona is disassembled into a spot-like KNL1/Mis12/Ndc80-distal domain (Roscioli et al., 2019), and spindle assembly checkpoint signaling is silenced. This is a result, in part, of the dynein-mediated stripping of Mad2 and Rod from kinetochores toward the minus ends of spindle microtubules (Howell et al., 2001; Siller et al., 2005; Wojcik et al., 2001). The contribution of CENP-F to these corona processes is less well understood.

Originally termed mitosin, CENP-F is a large (∼360-kD) coiled-coil protein that dimerizes and localizes to diverse subcellular locations, including microtubule plus-ends, mitochondria, nuclear pores, and kinetochores (Berto and Doye, 2018; Berto et al., 2018; Kanfer et al., 2015; Rattner et al., 1993). CENP-F contains several nonoverlapping functional domains, which include two high-affinity microtubule-binding domains (MTBDs), one at either terminus (Feng et al., 2006; Kanfer et al., 2017; Musinipally et al., 2013; Volkov et al., 2015), and binding sites for kinetochore (Bub1 [budding uninhibited by benzimidazoles 1]; Berto et al., 2018; Ciossani et al., 2018), mitochondrial (Miro [mitochondrial rho]; Kanfer et al., 2015; Kanfer et al., 2017; Peterka and Kornmann, 2019), and nuclear pore (Nup133 [nuclear pore 133]; Berto and Doye, 2018; Berto et al., 2018) adapttnycouer proteins. Both MTBDs have a similar microtubule-binding affinity to the major kinetochore attachment factor Ndc80 (Volkov et al., 2015). In vitro reconstitution experiments revealed that both MTBDs are able to autonomously track depolymerizing microtubule plus-ends in vitro, although the amino-terminal MTBD has a higher preference for curved protofilaments (Volkov et al., 2015). Therefore, the adapter-dependent recruitment of CENP-F to subcellular structures allows them to harness microtubule plus-end dynamics to do work. In line with this, a recent work showed that Miro-CENP-F couples mitochondria to dynamic microtubule tips (Kanfer et al., 2015; Kanfer et al., 2017; Peterka and Kornmann, 2019).

CENP-F recruitment to kinetochores does not involve the major corona complex RZZ-S but rather a direct interaction between a defined targeting domain (amino acids 2826–2894) and the kinase domain of Bub1 (Ciossani et al., 2018). Indeed, recruitment of CENP-F to kinetochores is severely compromised in the absence of Bub1 (Ciossani et al., 2018; Currie et al., 2018; Johnson et al., 2004; Liu et al., 2006; Raaijmakers et al., 2018). Furthermore, CENP-F is implicated in the recruitment of the molecular motors dynein (and the Nde1/Ndel1/Lis1 regulators) and CENP-E (Bomont et al., 2005; Faulkner et al., 2000; Simões et al., 2018; Stehman et al., 2007; Tai et al., 2002; Vergnolle and Taylor, 2007; Wynne and Vallee, 2018; Yang et al., 2005). Early RNAi studies reported that depletion of CENP-F is associated with severe chromosome congression defects (Bomont et al., 2005; Feng et al., 2006; Holt et al., 2005; Vergnolle and Taylor, 2007; Yang et al., 2005); however, CENP-F knockout mice are viable (Haley et al., 2019), and recent CRISPR knockouts in haploid human cells do not show chromosome alignment defects (Raaijmakers et al., 2018). Defining how CENP-F contributes to chromosome segregation processes thus remains unresolved.

## Results

### CENP-F MTBDs are required for interkinetochore (K-K) tension and stable microtubule attachment

**Figure 1.**
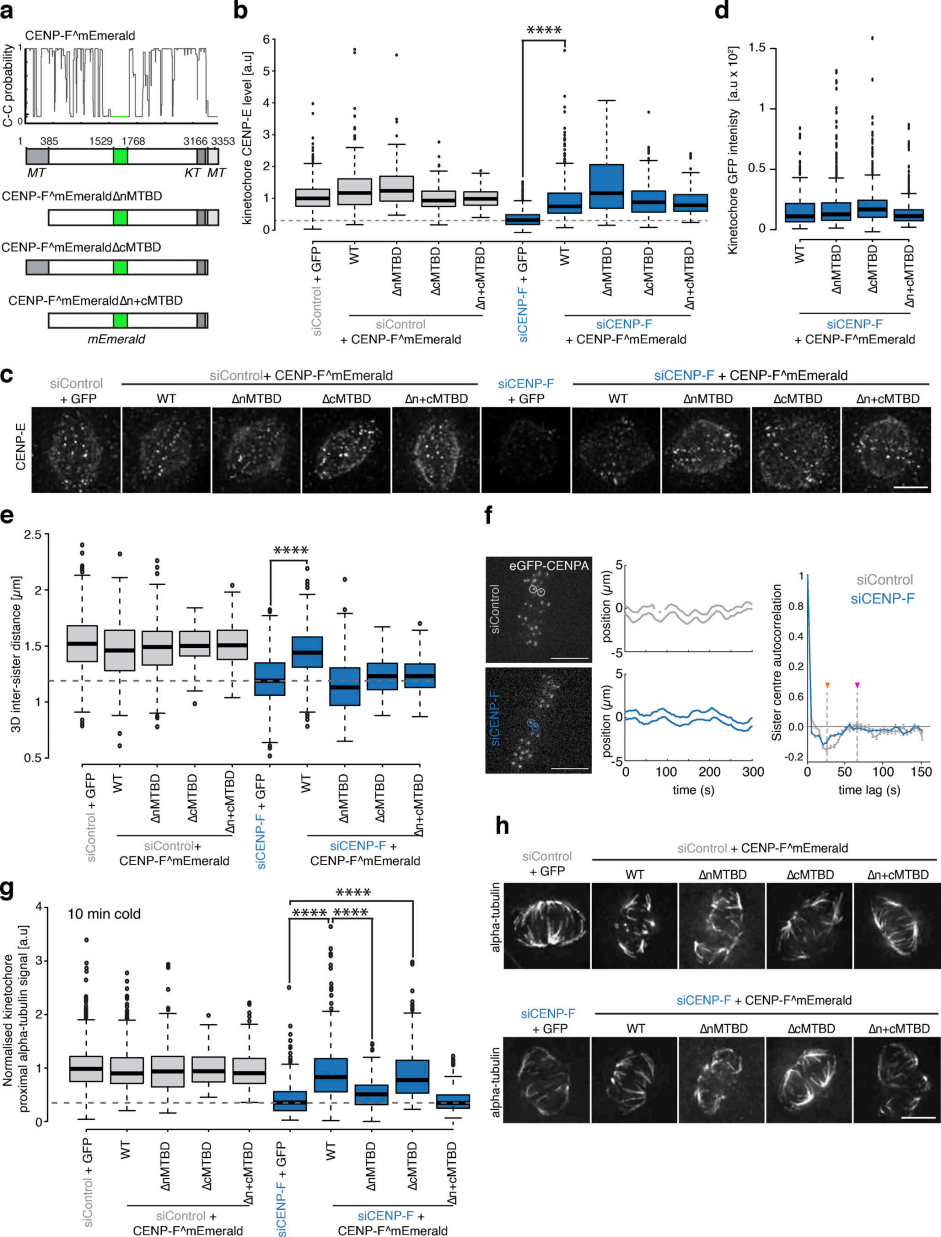
**CENP-F MTBDs are required for K-K tension and stable microtubule attachment. (a)** Top: Coiled-coil prediction of the CENP-F^mEmerald protein. Bottom: Schematic showing the position of the microtubule- and kinetochore-binding domains in CENP-F^mEmerald and mutant versions. **(b)** Quantification of kinetochore CENP-E intensity relative to CENP-C in the rescue experiment shown in c and Fig. S1 a. **(c)** Immunofluorescence microscopy images of HeLa-K cells treated with either control or CENP-F siRNA, transfected with CENP-F^mEmerald or a microtubule-binding mutant and stained with DAPI and antibodies against CENP-C and CENP-E. Scale bar, 5 µm. Only the CENP-E channel is shown. **(d)** Quantification of kinetochore eGFP intensity in HeLa-K cells transfected with either CENP-F^mEmerald or a microtubule-binding mutant. **(e)** Quantification of the CENP-C–based intersister distance in the CENP-F rescue experiment depicted in Fig. S1 b. Dotted gray line indicates the intersister distance in cells treated with CENP-F siRNA and transfected with an empty vector. **(f)** Quantification of kinetochore oscillatory movements in control and CENP-F siRNA–treated cells using KiT software (Olziersky et al., 2018). Left: Example images of eGFP-CENP-A–expressing HeLa cells and corresponding charts showing tracks from example sister pairs (circled). Right: Autocorrelation plot based on the oscillations of eGFP-CENP-A kinetochores. The orange and purple arrowheads indicate the half and full periods in control cells, respectively (control siRNA: 23 cells, 783 sisters; CENP-F siRNA, 33 cells, 1,137 sisters). Scale bar, 5 µm. **(g)** Quantification of the kinetochore proximal α-tubulin intensity relative to CENP-C in the cold-stable CENP-F rescue experiment depicted in panel h and Fig. S1 c. Dotted gray line indicates the α-tubulin intensity in cells treated with CENP-F siRNA and rescued with an empty vector. **(h)** Immunofluorescence microscopy images of the cold-stable CENP-F rescue experiment. HeLa-K cells were treated with either control or CENP-F siRNA and transfected with CENP-F^mEmerald or a microtubule-binding mutant before incubation on ice and fixation. Cells were stained with DAPI and antibodies against CENP-C and α-tubulin. Only the α-tubulin channel is shown. Scale bar, 5 µm. In b, d, e, and g, boxes depict the median and first and third quartiles, and whiskers represent Q1 and Q3 ± 1.5× interquartile range. ****, P < 0.0001.

**Figure S1.**
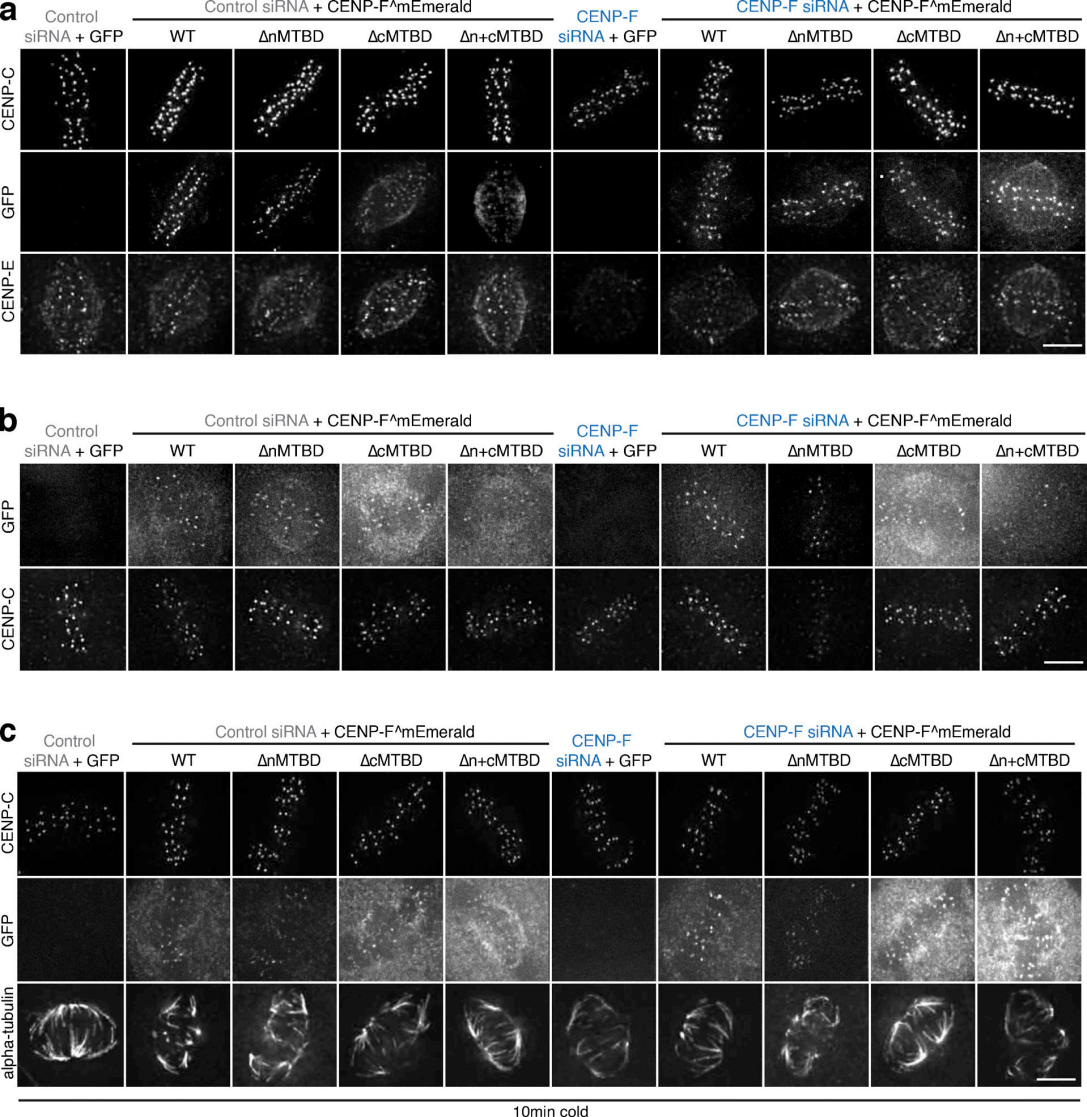
**CENP-F microtuuble binding mutant rescues.** **(a)** Immunofluorescence microscopy images of the CENP-E rescue experiment with CENP-F MTBD mutants. HeLa-K cells were treated with control or CENP-F siRNA and rescued with an empty vector, CENP-F^mEmerald, CENP-F^mEmeraldΔnMTBD, CENP-F^mEmeraldΔcMTBD, or CENP-F^mEmeraldΔn+cMTBD before being stained with DAPI and antibodies against CENP-C and CENP-E. Scale bar, 5 µm. **(b)** Immunofluorescence microscopy images of the K-K distance rescue experiment with CENP-F MTBD mutants. HeLa-K cells were treated with control or CENP-F siRNA and rescued with an empty vector, CENP-F^mEmerald, CENP-F^mEmeraldΔnMTBD, CENP-F^ΔcMTBD, or CENP-F^GFPΔn+cMTBD before being stained with DAPI and an antibody against CENP-C. Scale bar, 5 µm. **(c)** Immunofluorescence microscopy images of the cold-stable rescue experiment with CENP-F MTBD mutants. HeLa-K cells were treated with control or CENP-F siRNA and rescued with an empty vector, CENP-F^mEmerald, CENP-F^GFPΔnMTBD, CENP-F^GFPΔcMTBD, or CENP-F^GFPΔn+cMTBD before being incubated on ice for 10 min and stained with DAPI and antibodies against CENPC and α-tubulin. Scale bar, 5 µm.

Given the reported pleiotropic phenotypes associated with CENP-F RNAi experiments (Bomont et al., 2005; Feng et al., 2006; Holt et al., 2005; Kanfer et al., 2015; Peterka and Kornmann, 2019; Vergnolle and Taylor, 2007; Yang et al., 2005), we reasoned that the two CENP-F MTBDs might make distinct contributions to kinetochore function. We therefore constructed CENP-F transgenes (a kind gift from B. Kornmann, University of Oxford, Oxford, UK) that have an mEmerald inserted at position 1529 between two predicted coiled-coils and either one or both of the MTBDs deleted (see schematic in Fig. 1 a). First, we confirmed previous work showing that CENP-E motors were lost (by ∼50%) from kinetochores in CENP-F siRNA–treated cells (Yang et al., 2005; Fig. 1, b and c; and Fig. S1 a; *n* ≥ 2, 150 KTs/15 cells per condition). Expression of wild-type and mutant CENP-F transgenes restored CENP-E localization (Fig. 1, b and c; and Fig. S1 a; *n* ≥ 2, 150 KTs/15 cells per condition) and bound kinetochores to the same extent (Figs. 1 d and S1 a; *n* ≥ 3, 515 KTs/52 cells per condition). The K-K distance between CENP-C signals was also reduced from 1.49 ± 0.23 µm in control cells to 1.2 ± 0.22 µm in CENP-F–depleted cells (Figs. 1 e and S1 b; *n* ≥ 3, 230 KTs/23 cells per condition; Bomont et al., 2005). Consistent with the loss of K-K tension, we found that depletion of CENP-F also perturbs the normal semiperiodic oscillations of sister kinetochores in metaphase (Fig. 1 f). Transfection with full-length CENP-F^mEmerald rescued the K-K distance to 1.44 ± 0.22 µm. In contrast, the CENP-F^mEmeraldΔnMTBD (missing amino-terminal MTBD), CENP-F^mEmeraldΔcMTBD (missing carboxy-terminal MTBD), or CENP-F^mEmeraldΔn+cMTBD (both MTBDs deleted) were not able to rescue this phenotype (Figs. 1 e and S1 b; *n* ≥ 3, 230 KTs/23 cells per condition).

Because K-K distance is dependent on the pulling forces generated by microtubule attachment, we tested the stability of kinetochore-microtubule attachments. To do so, we incubated cells on ice for 10 min before fixation and then quantified the kinetochore-proximal α-tubulin signal as a readout of microtubule number. In CENP-F siRNA–treated cells transfected with an empty vector, the α-tubulin intensities were reduced to 41 ± 28% compared with control (Fig. 1, g and h; and Fig. S1 c; *n* ≥ 3, 270 KTs/27 cells per condition). Transfection with CENPF^mEmerald or CENP-F^mEmeraldΔcMTBD rescued α-tubulin levels to 88 ± 49% and 85 ± 45%, respectively (Fig. 1, g and h; and Fig. S1 c; *n* ≥ 3, 270 KTs/27 cells per condition). In contrast, cells expressing either CENP-F^mEmeraldΔnMTBD or CENP-F^mEmeraldΔn+cMTBD failed to restore microtubule stability (α-tubulin intensities of 49 ± 26% and 37 ± 18%), demonstrating that the CENP-F N-terminal MTBD is necessary for cold-stable microtubule attachment (Fig. 1, g and h; and Fig. S1 c; *n* ≥ 3, 270 KTs/27 cells per condition). Taken together, these separation-of-function mutants show how microtubule binding by CENP-F is required for stable microtubule-kinetochore attachment and the generation of normal tension across sister kinetochores, but dispensable for the normal localization of CENP-E motors.

### CENP-F CRISPR mutants phenocopy CENP-F RNAi

**Figure 2.**
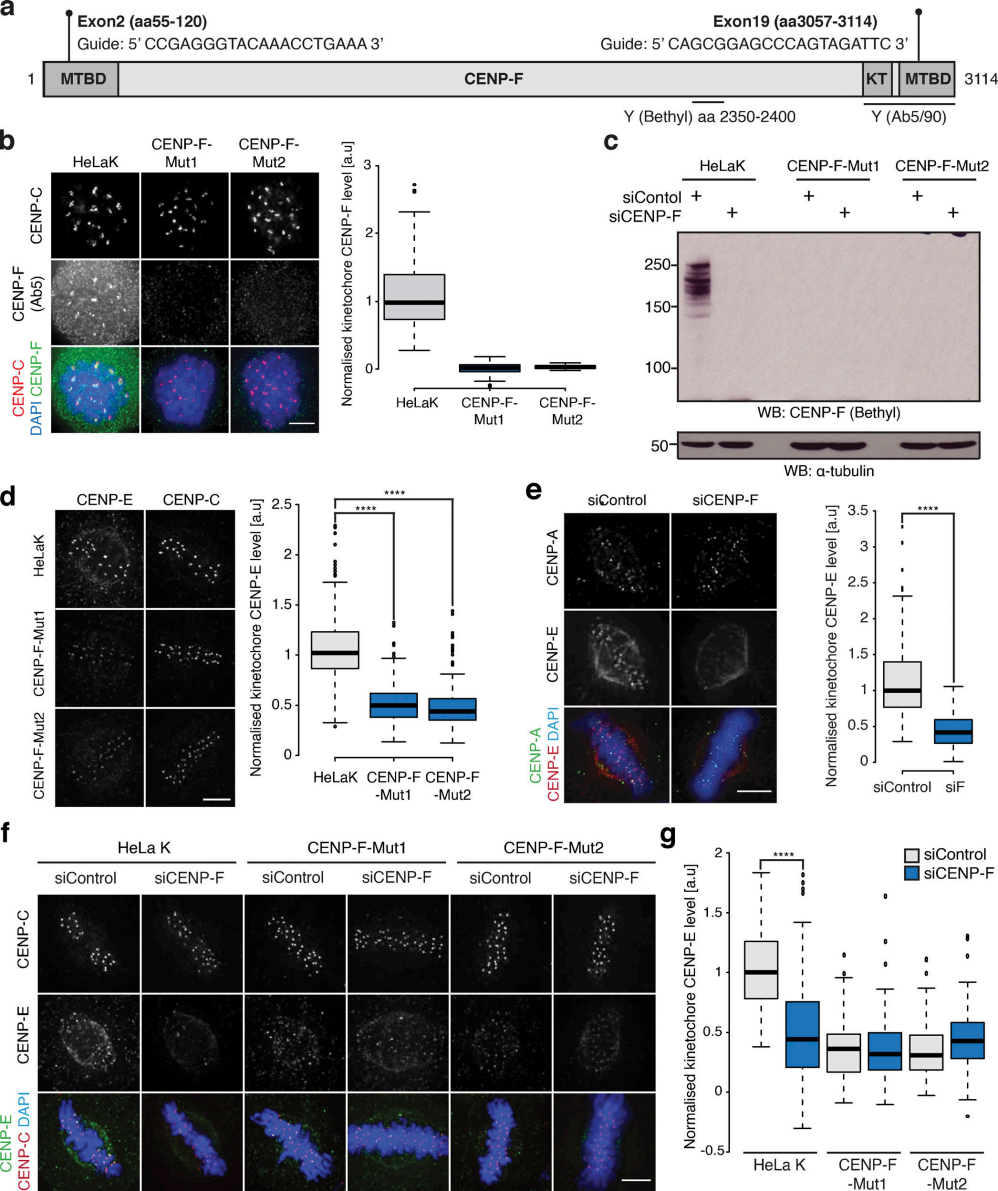
**CENP-F CRISPR mutants phenocopy CENP-F RNAi. (a)** Schematic showing the CRISPR guide targets and antibody epitopes in CENP-F. **(b)** Left: Immunofluorescence microscopy images of HeLa-K, CENP-F-Mut1, and CENP-F-Mut2 cells arrested in 330 nM nocodazole for 16 h and stained with antibodies against CENP-C and CENP-F (Ab5). Scale bar, 5 µm. Right: Quantification of kinetochore CENP-F(Ab5) intensity relative to CENP-C in HeLa-K, CENP-F-Mut1, and CENP-F-Mut2 cells arrested in 330 nM nocodazole for 16 h. **(c)** Immunoblot of liquid-N_2_ extracts collected from HeLa-K, CENP-F-Mut1, and CENP-F-Mut2 cells treated with either control or CENP-F siRNA and arrested in 330 nM nocodazole for 16 h. The membrane was probed with antibodies against CENP-F(Bethyl) and α-tubulin. **(d)** Left: Immunofluorescence microscopy images of HeLa-K, CENP-F-Mut1, and CENP-F-Mut2 cells stained with DAPI and antibodies against CENP-E and CENP-C. Scale bar, 5 µm. Right: Quantification of kinetochore CENP-E intensity relative to CENP-C in HeLa-K, CENP-F-Mut1, and CENP-F-Mut2 cells. **(e)** Left: Immunofluorescence microscopy images of HeLa-K cells treated with either control or CENP-F siRNA and stained with DAPI and antibodies against CENP-E and CENP-A. Scale bar, 5 µm. Right: Quantification of kinetochore CENP-E intensity relative to CENP-A in cells treated with either control or CENP-F siRNA. **(f)** Immunofluorescence microscopy images of HeLa-K, CENP-F-Mut1, and CENP-F-Mut2 cells treated with either control or CENP-F siRNA and stained with DAPI and antibodies against CENP-E and CENP-C. Scale bar, 5 µm. **(g)** Quantification of kinetochore CENP-E level relative to CENP-C in HeLa-K, CENP-F-Mut1, and CENP-F-Mut2 cells treated with control or CENP-F siRNA. In b, d, e, and g, boxes depict the median and first and third quartiles, and whiskers represent Q1 and Q3 ± 1.5× interquartile range. ****, P < 0.0001.

**Figure S2.**
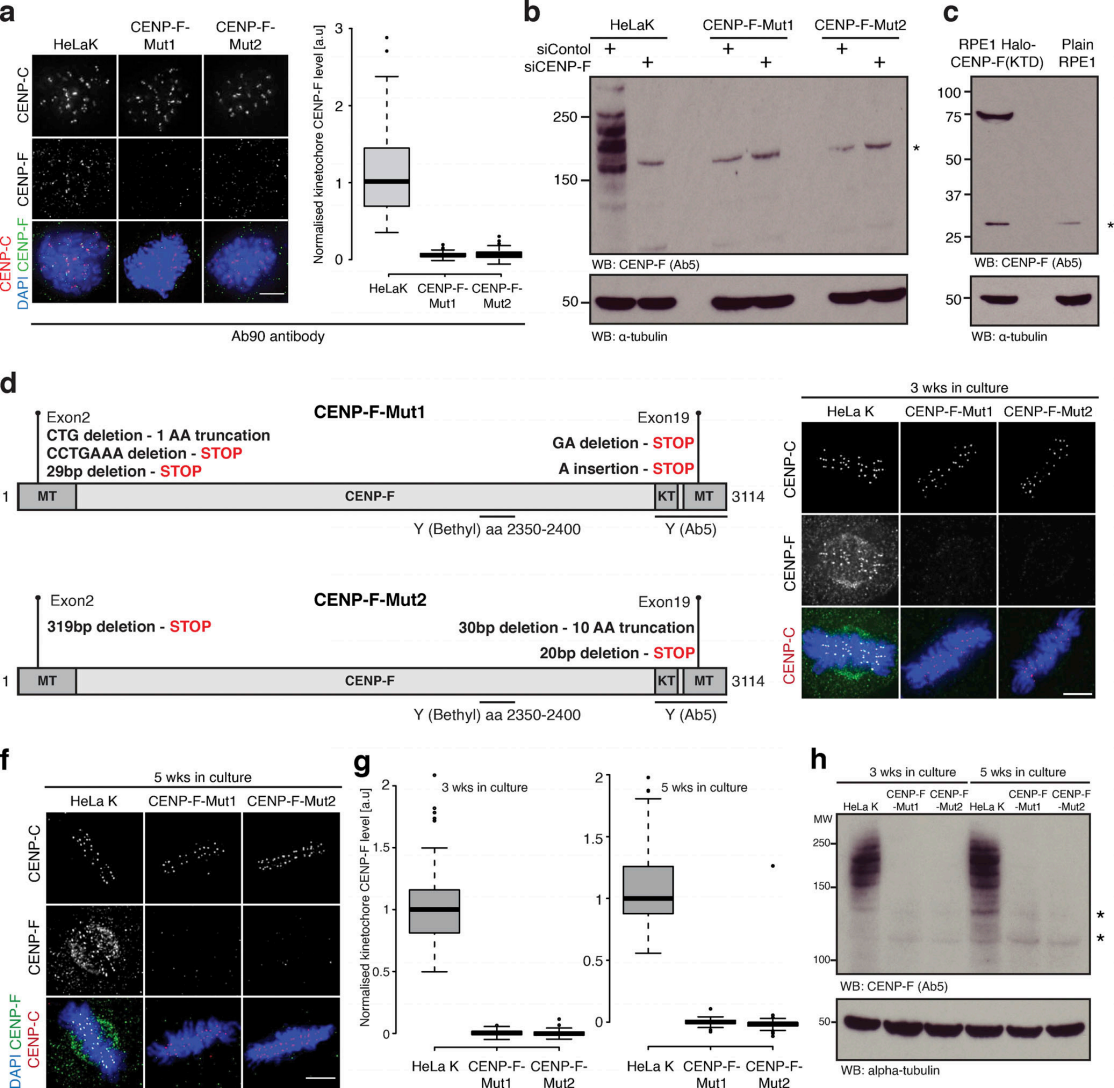
**Characterisation of CENP-F-Mut1 and CENP-F-Mut2 cells.** **(a)** Left: Immunofluorescence microscopy images of HeLa-K, CENP-F-Mut1, and CENP-FMut2 cells treated with 330 nM nocodazole for 16 h and stained with DAPI and antibodies against CENP-C and CENP-F(Ab90). Scale bar, 5 µm. Right: Quantification of kinetochore CENP-F intensity relative to CENP-C in HeLa-K, CENP-F-Mut1, and CENP-F-Mut2 cells arrested in 330 nM nocodazole for 16 h. **(b)** Immunoblot of liquid N2 protein extracts from HeLa-K, CENP-F-Mut1, and CENP-F-Mut2 cells treated with either control or CENP-F siRNA and arrested in nocodazole for 16 h. The membrane was probed with antibodies against CENP-F(Ab5) and α-tubulin. Asterisk indicates a nonspecific band. **(c)** Immunoblot of liquid N2 protein extracts from Rpe1 and Rpe1-Halo-CENP-F(KTD) cells arrested in nocodazole for 16 h. The membrane was probed with antibodies against CENP-F(Ab5) and α-tubulin. Asterisk indicates a nonspecific band. **(d)** Summary of CENP-F-Mut1 and CENP-F-Mut2 exon2 and exon19 sequencing. **(e)** Immunofluorescence microscopy images of HeLa-K, CENP-F-Mut1, and CENP-F-Mut2 cells cultured for 3 wk and stained with DAPI and antibodies against CENP-C and CENP-F (Ab5). Scale bar, 5 µm. **(f)** Immunofluorescence microscopy images of HeLa-K, CENP-F-Mut1, and CENP-F-Mut2 cells cultured for 5 wk and stained with DAPI and antibodies against CENP-C and CENP-F (Ab5). Scale bar, 5 µm. **(g)** Quantification of kinetochore CENP-F intensity relative to CENP-C in HeLa-K, CENP-F-Mut1, and CENP-F-Mut2 cells cultured for 3 (left) or 5 (right) wk. **(h)** Immunoblot of liquid N2 protein extracts from HeLa-K, CENP-F-Mut1, and CENP-F-Mut2 cells grown in culture for 3 or 5 wk and arrested in 330 nM nocodazole for 16 h. Membranes were probed with antibodies against CENP-F (Ab5) and α-tubulin. In a and g, boxes depict the median and first and third quartiles, and whiskers represent Q1 and Q3 ± 1.5× interquartile range. Asterisk indicates nonspecific bands.

**Figure S3.**
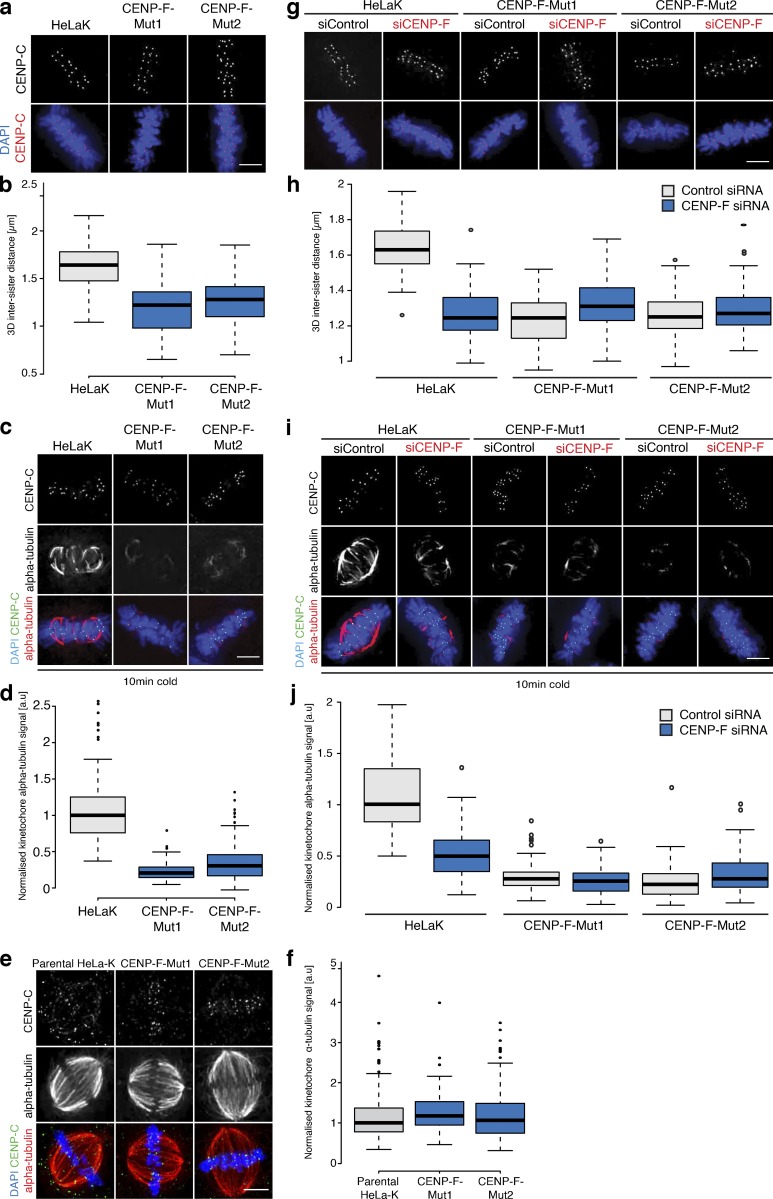
**CENP-F CRISPR mutants phenocopy CENP-F siRNA.** **(a)** Immunofluorescence microscopy images of HeLa-K, CENP-F-Mut1, and CENP-F-Mut2 cells stained with DAPI and an antibody against CENP-C. Scale bar, 5 µm. **(b)** Quantification of the CENP-C–based intersister distance in HeLa-K, CENP-F-Mut1, and CENP-F-Mut2 cells. **(c)** Immunofluorescence microscopy images of HeLa-K, CENP-F-Mut1, and CENP-F-Mut2 cells incubated on ice for 10 min before being stained with DAPI and antibodies against CENP-C and α-tubulin. Scale bar, 5 µm. **(d)** Quantification of kinetochore proximal α-tubulin intensity in HeLa-K, CENP-F-Mut1, and CENP-F-Mut2 cells incubated on ice for 10 min. **(e)** Immunofluorescence microscopy images of untreated and glutaraldehyde-fixed HeLa-K, CENP-F-Mut1, or CENP-F-Mut2 cells stained with DAPI and antibodies against α-tubulin and CENP-C. Scale bar, 5 µm. **(f)** Quantification of kinetochore proximal α-tubulin intensity in untreated and glutaraldehyde-fixed HeLa-K, CENP-F-Mut1, or CENP-F-Mut2 cells. **(g)** Immunofluorescence microscopy images of HeLa-K, CENP-F-Mut1, and CENP-F-Mut2 cells treated with either control or CENP-F siRNA and stained with DAPI and an antibody against CENP-C. Scale bar, 5 µm. **(h)** Quantification of the CENP-C–based intersister distance in HeLa-K, CENP-FMut1, and CENP-F-Mut2 cells treated with either control or CENP-F siRNA. **(i)** Immunofluorescence microscopy images of HeLa-K, CENP-F-Mut1, and CENP-F-Mut2 cells treated with control or CENP-F siRNA, incubated on ice for 10 min, and stained with DAPI and antibodies against CENP-C and α-tubulin. Scale bar, 5 µm. **(j)** Quantification of kinetochore proximal α-tubulin intensity in HeLa-K, CENP-F-Mut1, and CENP-F-Mut2 cells treated with control or CENP-F siRNA and incubated on ice for 10 min. In b, d, f, h, and j, boxes depict the median and first and third quartiles, and whiskers represent Q1 and Q3 ± 1.5× interquartile range.

Our data show that CENP-F is required for the localization of a subset (∼50%) of CENP-E motors to kinetochores (Fig. 1, b and c). To rule out partial RNAi depletion effects, we generated mutant cell lines by simultaneously targeting CENP-F exons 2 and 19 using CRISPR/Cas9 (Fig. 2 a; Raaijmakers et al., 2018) and obtained two independent clonal lines (named CENP-F-Mut1 and CENP-F-Mut2; Figs. 2 and S2). In both clones, we recovered only alleles with premature stop codons (Fig. S2 d) and were unable to detect CENP-F protein by quantitative immunofluorescence and Western blotting using antibodies against two different C-terminal epitopes (Fig. 2, b and c; and Fig. S2, a and b; *n* = 1, 100 KTs/10 cells per condition). We note that the Ab5 epitope is partly encoded by exon 19 and could therefore be destroyed by repair of the double strand break. To rule this out, we prepared protein extracts from Rpe1 cells stably expressing the CENP-F kinetochore-targeting domain (KTD; fused to Halo-Tag), which is located downstream of the cut site (Fig. 2 a), and blotted for CENP-F using the Ab5 antibody. As shown in Fig. S2 c, the KTD fragment was clearly detected, confirming that the Ab5 antibody can recognize an epitope downstream of exon 19. We also confirmed that long-term passage of these lines did not lead to any (detectable) reexpression of CENP-F, which would be consistent with CENP-F being nonessential in human cells (Fig. S2, e–h; *n* = 1, 100 KTs/10 cells per condition). CENP-E levels on metaphase kinetochores were reduced to 47 ± 18% and 45 ± 19% in CENP-F-Mut1 and CENP-F-Mut2 cells, respectively (Fig. 2 d; *n* = 3, 300 KTs/30 cells per condition), equivalent to measurements in our RNAi experiments (Fig. 1, b and c; and Fig. 2 e). These cells also displayed a reduction in K-K distance and had fewer cold-stable microtubules (Fig. S3, a–d; *n* = 2, 200 KTs/20 cells per condition). The latter was not due to perturbation of K-fiber formation, as the kinetochore-proximal α-tubulin intensity in untreated and glutaraldehyde-fixed parental, CENP-F-Mut1 and CENP-F-Mut2 cells was comparable (Fig. S3, e and f; *n* = 2, 200 KTs/20 cells per condition). Because trace amounts of functional proteins can remain in CRISPR clones (Meraldi, 2019), we repeated these analyses in CENP-F-Mut1 and CENP-F-Mut2 cells treated with CENP-F siRNA. We did not detect any additive phenotypes, providing further evidence that the CENP-F mutants are loss-of-function alleles and that the siRNA is both on target and efficient at depleting CENP-F (Fig. 2, f and g; and Fig. S3, g–j; *n* = 1, 100 KTs/10 cells per condition).

### CENP-F influences corona composition in a microtubule-dependent manner

**Figure 3.**
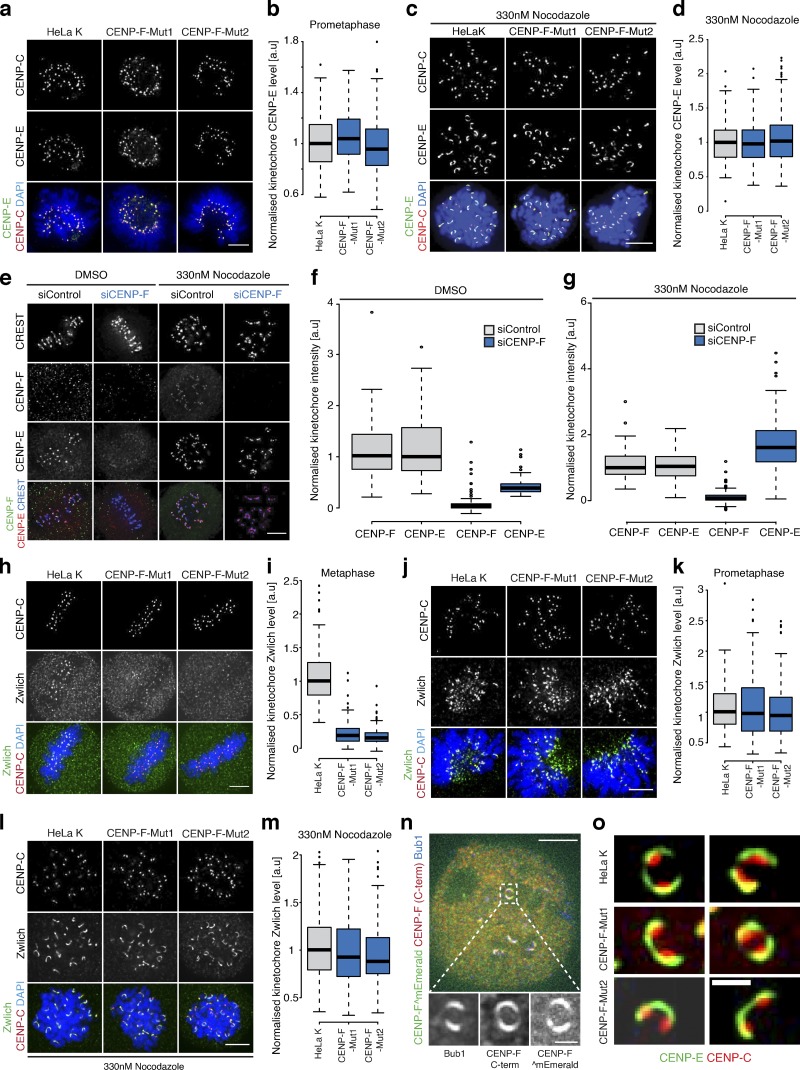
**CENP-F controls corona localization in a microtubule-dependent manner. (a)** Immunofluorescence microscopy images of early prometaphase HeLa-K, CNP-F-Mut1, and CENP-F-Mut2 cells stained with DAPI and antibodies against CENP-C and CENP-E. Scale bar, 5 µm. **(b)** Quantification of kinetochore CENP-E intensity relative to CENP-C in early prometaphase HeLa-K, CENP-F-Mut1, and CENP-F-Mut2 cells. **(c)** Immunofluorescence microscopy images of HeLa-K, CENP-F-Mut1, and CENP-Mut2 treated with 330 nM nocodazole for 16 h and stained with DAPI and antibodies against CENP-C and CENP-E. Scale bar, 5 µm. **(d)** Quantification of kinetochore CENP-E intensities relative to CENP-C in HeLa-K, CENP-F-Mut1, and CENP-Mut2 treated with 330 nM nocodazole for 16 h. **(e)** Immunofluorescence microscopy images of HeLa-K cells treated with control or CENP-F siRNA, incubated with DMSO or 330 nM nocodazole for 16 h and stained with DAPI, CREST antisera and antibodies against CENP-E and CENP-F. Scale bar, 5 µm. The CREST display intensities are not comparable between DMSO and nocodazole conditions. **(f)** Quantification of kinetochore CENP-E and CENP-F levels relative to CREST in HeLa-K cells treated with control or CENP-F siRNA and incubated with DMSO for 16 h. **(g)** Quantification of kinetochore CENP-E and CENP-F levels relative to CREST in HeLa-K cells treated with control or CENP-F siRNA and incubated with 330 nM nocodazole for 16 h. **(h)** Immunofluorescence microscopy images of metaphase HeLa-K, CENP-F-Mut1, and CENP-F-Mut2 cells stained with antibodies against CENP-C and Zwlich. Scale bar, 5 µm. **(i)** Quantification of kinetochore Zwlich level relative to CENP-C at kinetochores in metaphase HeLa-K, CENP-F-Mut1, and CENP-F-Mut2 cells. **(j)** Immunofluorescence microscopy images of early prometaphase HeLa-K, CENP-F-Mut1, and CENP-F-Mut2 cells stained with antibodies against CENP-C and Zwlich. Scale bar, 5 µm. **(k)** Quantification of kinetochore Zwlich intensities relative to CENP-C in prometaphase HeLa-K, CENP-F-Mut1, and CENP-F-Mut2 cells. **(l)** Immunofluorescence microscopy images of HeLa-K, CENP-F-Mut1, and CENP-Mut2 cells treated with 330 nM nocodazole for 16 h and stained with antibodies against CENP-C and Zwlich. Scale bar, 5 µm. **(m)** Quantification of kinetochore Zwlich intensities relative to CENP-C in HeLa-K, CENP-F-Mut1, and CENP-Mut2 cells treated with 330 nM nocodazole for 16 h. **(n)** Immunofluorescence microscopy images of HeLa-K cells transfected with CENP-F^mEmerald, arrested in 330 nM nocodazole for 16 h, and stained with antibodies against Bub1 and CENP-F C-terminus (Ab5). Insets show a zoom of an expanded kinetochore. Scale bars, 5 µm (upper); 1 µm (lower). **(o)** Immunofluorescence microscopy images of CENP-E crescents in HeLa-K, CENP-F-Mut1, and CENP-F-Mut2 cells arrested in 330 nM nocodazole for 16 h. Scale bar, 1µm. In b, d, f, g, i, k, and m, boxes depict the median and first and third quartiles, and whiskers represent Q1 and Q3 ± 1.5× interquartile range.

It is well established that components of the corona are shed from kinetochores as end-on attachments form and biorientation is achieved (Bancroft et al., 2015; Gassmann et al., 2010; Hoffman et al., 2001; Magidson et al., 2015; Maiato et al., 2004; Pereira et al., 2018; Sacristan et al., 2018; Thrower et al., 1996). This raises the possibility that the binding/unbinding of corona components may be sensitive to microtubule attachment status. To test this, we first quantified the CENP-E intensity at early prometaphase kinetochores in CENP-F-Mut1 and CENP-F-Mut2 cells, a time when sister pairs lack end-on attachment and are predominantly unattached or laterally attached (Magidson et al., 2011). This revealed that CENP-E was fully loaded (Fig. 3, a and b; *n* = 2, 200 KTs/20 cells per condition), even in the absence of CENP-F. To further this observation, we treated parental and CENP-F-Mut1 or -Mut2 clones with nocodazole and again found that CENP-E motors relocalized to kinetochores (to 99.7 ± 29.1% and 103.2 ± 35%, respectively; Fig. 3, c and d; *n* = 2, 200 KTs/20 cells per condition). We could also confirm this result using CENP-F siRNA–treated cells (38 ± 13.8% in DMSO vs. 161.7 ± 100.1% in nocodazole; Fig. 3, e–g; *n* = 1, 100 KTs/10 cells per condition). We next tested whether this behavior was common to other corona components. In line with our CENP-E data, Zwlich (RZZ complex subunit) was reduced to 20.4 ± 15.4% and 17.3 ± 12.1% at metaphase kinetochores in CENP-F-Mut1 and CENP-F-Mut2 cells, respectively (Fig. 3, h and i; *n* = 2, 200 KTs/20 cells per condition). Moreover, Zwlich localization was comparable to control in early prometaphase (102 ± 49% and 97 ± 42%; *n* = 2, 200 KTs/20 cells per condition) or nocodazole-treated cells (95.2 ± 33.3% and 93.8 ± 31.3%; *n* = 2, 200 KTs/20 cells per condition; Fig. 3, j–m). This demonstrates how CENP-F influences the localization of multiple corona components after end-on microtubule attachment, a finding consistent with previous observations (Yang et al., 2005). In line with this, both the carboxy-terminus and central regions of CENP-F are part of crescent structures at unattached kinetochores (Fig. 3 n). However, CENP-F is not required for outer kinetochore expansion per se, as CENP-E crescents were clearly visible in both CENP-F-Mut1 and CENP-F-Mut2 clones (Fig. 3 o).

### CENP-F protects the corona from excessive dynein-mediated stripping

**Figure 4.**
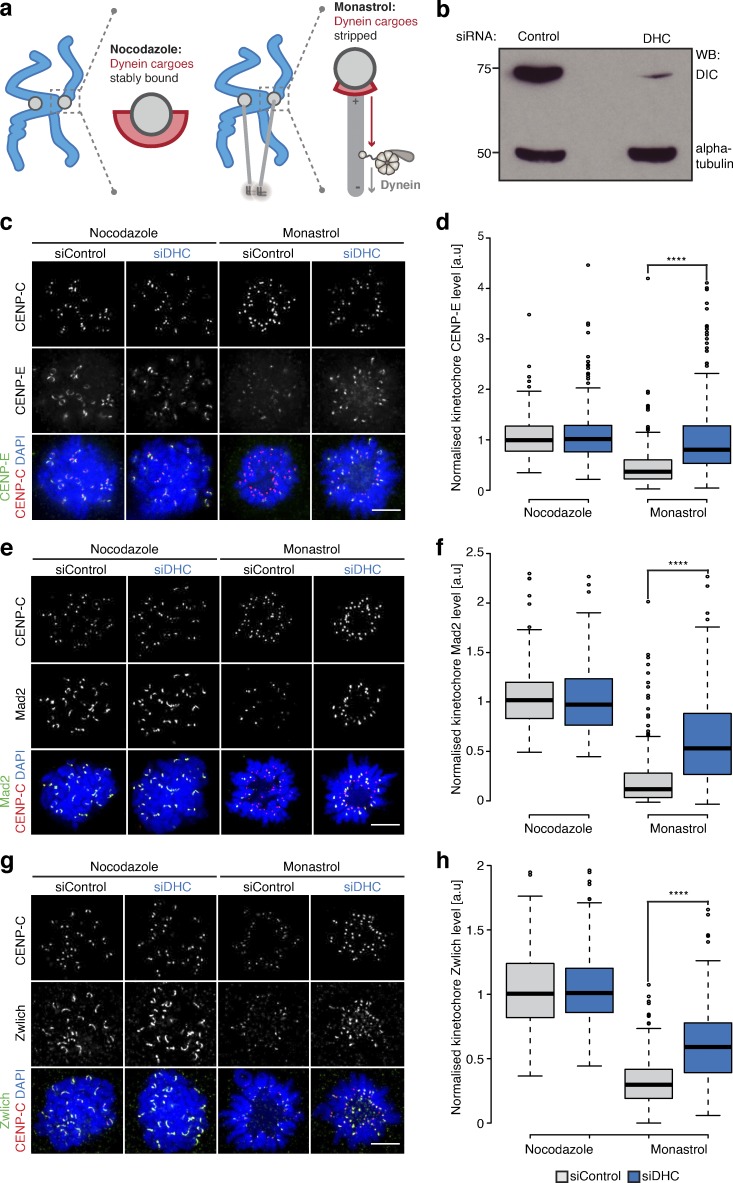
**Quantitative assay for dynein-mediated stripping. (a)** Schematic outlining the dynein-stripping assay. Briefly, we compare cells arrested in nocodazole, where dynein cargoes are stably bound, and monastrol, where dynein can transport cargoes away from the syntelic (end-on attached) kinetochores toward the monopole. **(b)** Immunoblot of liquid-N_2_ extracts collected from HeLa-K cells treated with control or DHC siRNA. The membrane was probed with antibodies against DIC and α-tubulin. **(c)** Immunofluorescence microscopy images from the dynein-stripping assay for CENP-E. Cells were treated with control or DHC siRNA, arrested in nocodazole or monastrol, and stained with DAPI and antibodies against CENP-E and CENP-C. Scale bar, 5 µM. **(d)** Quantification of the kinetochore CENP-E intensity relative to CENP-C in the dynein-stripping assay as depicted in c. **(e)** Immunofluorescence microscopy images of the dynein-stripping assay for Mad2. Cells were treated with control or DHC siRNA, arrested with nocodazole or monastrol, and stained with DAPI and antibodies against Mad2 and CENP-C. Scale bar, 5 µM. **(f)** Quantification of the kinetochore-bound Mad2 intensity relative to CENP-C in the dynein-stripping assay depicted in e. **(g)** Immunofluorescence microscopy images of the dynein-stripping assay for Zwlich. Cells were treated with control or DHC siRNA, arrested with nocodazole or monastrol, and stained with DAPI and antibodies against Zwlich and CENP-C. Scale bar, 5 µM. **(h)** Quantification of the kinetochore Zwlich intensity relative to CENP-C in the dynein-stripping assay as depicted in g. In d, f, and h, boxes depict the median and first and third quartiles, and whiskers represent Q1 and Q3 ± 1.5× interquartile range. ****, P < 0.0001.

**Figure S4.**
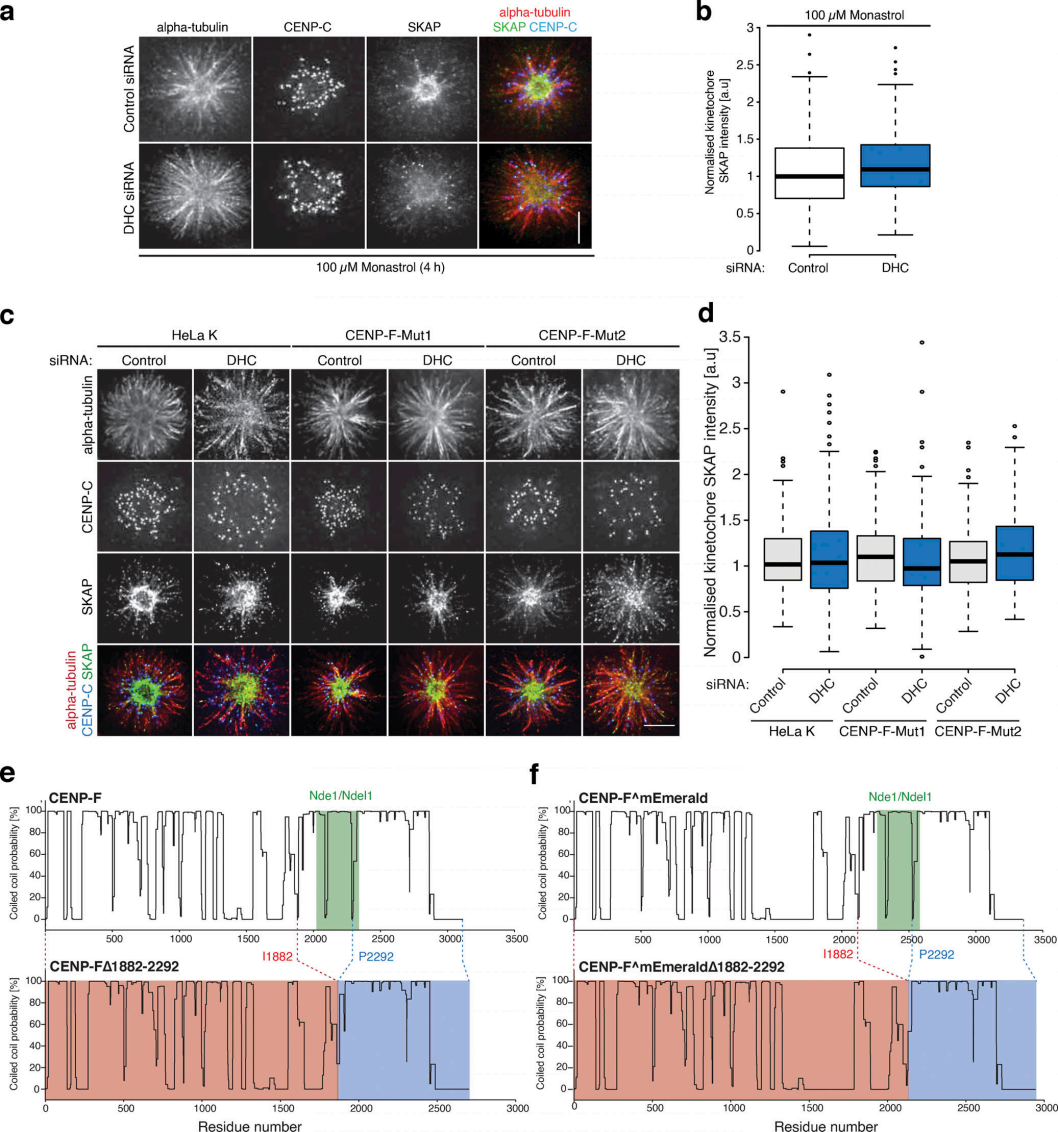
**SKAP controls for stripping assay and coiled-coil alignments for CENP-FΔ1882-2292.** **(a)** Immunofluorescence microscopy images of HeLa-K cells treated with control or DHC siRNA, arrested in 100 µM monastrol for 4 h, and stained with antibodies against CENP-C, α-tubulin, and SKAP. Scale bar, 5 µm. **(b)** Quantification of kinetochore SKAP signal relative to CENP-C in HeLa-K cells treated with control or DHC siRNA and arrested in 100 µM monastrol for 4 h. **(c)** Immunofluorescence microscopy images of HeLa-K, CENP-F-Mut1, and CENP-F-Mut2 cells treated with control or DIC siRNA, arrested in 100 µM monastrol for 4 h, and stained with antibodies against CENP-C, α-tubulin, and SKAP. Scale bar, 5 µm. **(d)** Quantification of kinetochore SKAP signal relative to CENP-C in HeLa-K, CENP-F-Mut1, and CENP-F-Mut2 cells treated with control or DIC siRNA and arrested in 100 µM monastrol for 4 h. **(e)** COILS (https://embnet.vital-it.ch/software/COILS_form.html) coiled-coil prediction for CENP-F and CENP-FΔ1882–2292. **(f)** COILS coiled-coil prediction for CENP-F^mEmerald and CENP-FΔ1882–2292^mEmerald. In b and d, boxes depict the median and first and third quartiles, and whiskers represent Q1 and Q3 ± 1.5× interquartile range.

**Figure 5.**
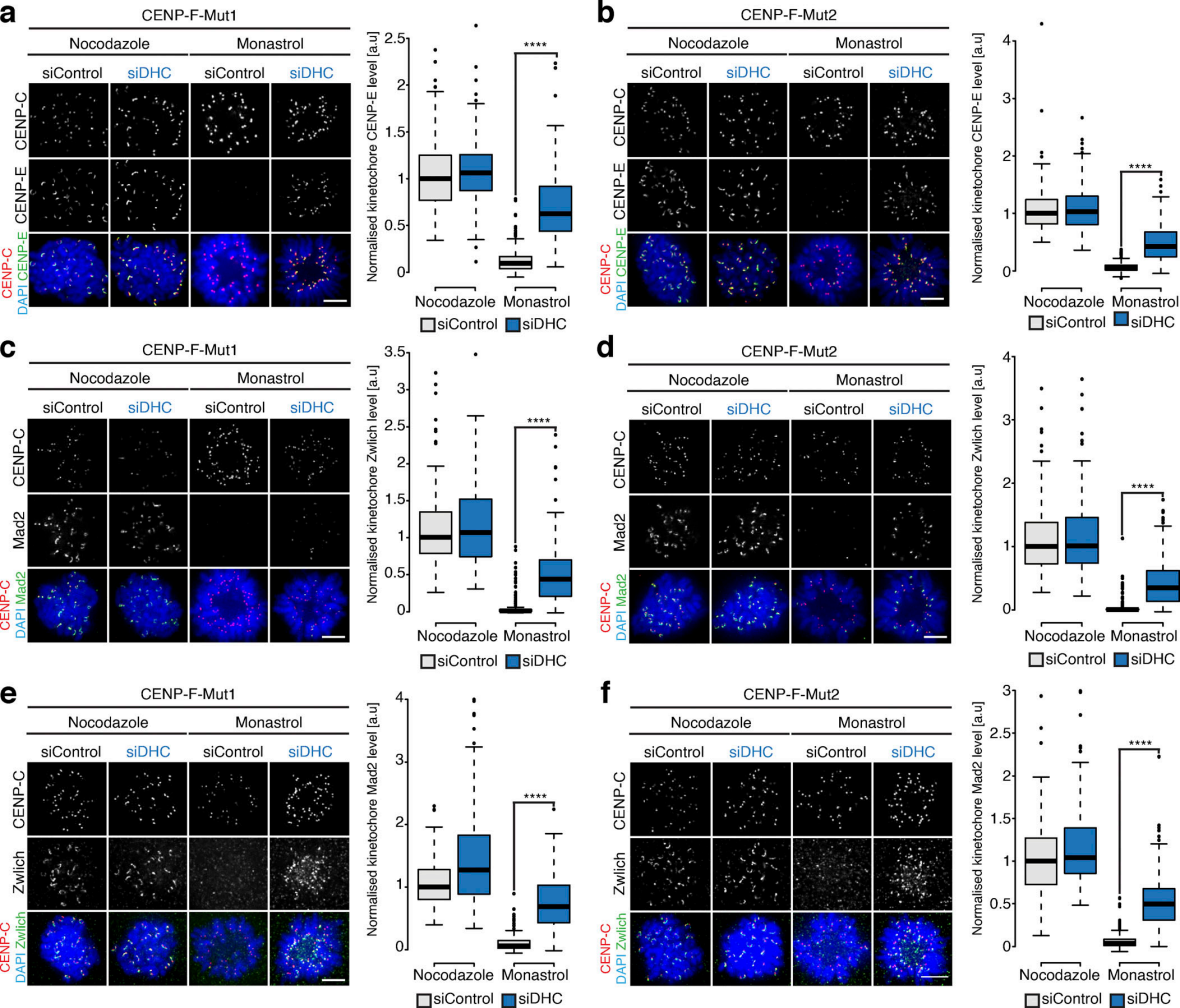
**CENP-F functions to limit dynein stripping of the corona. (a)** Left: Immunofluorescence microscopy images from the dynein-stripping assay for CENP-E in CENP-F-Mut1 cells. Cells were treated with control or DHC siRNA, arrested in nocodazole or monastrol, and stained with DAPI and antibodies against CENP-E and CENP-C. Scale bar, 5 µm. Right: Quantification of the kinetochore CENP-E intensity relative to CENP-C in the dynein-stripping assay. **(b)** Left: Immunofluorescence microscopy images of the dynein-stripping assay for CENP-E in CENP-F-Mut2 cells. Cells were treated with control or DHC siRNA, arrested in nocodazole or monastrol, and stained with DAPI and antibodies against CENP-E and CENP-C. Scale bar, 5 µm. Right: Quantification of the kinetochore CENP-E intensity relative to CENP-C in the dynein-stripping assay. **(c)** Left: Immunofluorescence microscopy images of the dynein-stripping assay for Mad2 in CENP-F-Mut1 cells. Cells were treated with control or DHC siRNA, arrested in nocodazole or monastrol, and stained with DAPI and antibodies against Mad2 and CENP-C. Scale bar, 5 µm. Right: Quantification of the kinetochore Mad2 intensity relative to CENP-C in the dynein-stripping assay. **(d)** Left: Immunofluorescence microscopy images of the dynein-stripping assay for Mad2 in CENP-F-Mut2 cells. Cells were treated with control or DHC siRNA, arrested in nocodazole or monastrol, and stained with DAPI and antibodies against Mad2 and CENP-C. Scale bar, 5 µm. Right: Quantification of the kinetochore Mad2 intensity relative to CENP-C in the dynein-stripping assay. **(e)** Left: Immunofluorescence microscopy images of the dynein-stripping assay for Zwlich in CENP-F-Mut1 cells. Cells were treated with control or DHC siRNA, arrested in nocodazole or monastrol, and stained with DAPI and antibodies against Zwlich and CENP-C. Scale bar, 5 µm. Right: Quantification of the kinetochore Zwlich intensity relative to CENP-C in the dynein-stripping assay. **(f)** Left: Immunofluorescence microscopy images of the dynein-stripping assay for Zwlich in CENP-F-Mut2 cells. Cells were treated with control or DHC siRNA, arrested in nocodazole or monastrol, and stained with DAPI and antibodies against Zwlich and CENP-C. Scale bar, 5 µm. Right: Quantification of the kinetochore Zwlich intensity relative to CENP-C in the dynein-stripping assay. Boxes depict the median and first and third quartiles, and whiskers represent Q1 and Q3 ± 1.5× interquartile range. ****, P < 0.0001.

The CENP-F–independent localization of CENP-E and Zwilch to unattached/lateral kinetochores suggests that CENP-F influences their metaphase localization indirectly. One possibility is that these molecules are stripped by dynein after microtubule attachment and that CENP-F modulates this stripping. This idea fits previous results showing how components of the corona are dynein cargoes (Howell et al., 2001) and CENP-F recruiting the Nde1-Ndel1-Lis1 dynein regulators to kinetochores (Faulkner et al., 2000; Siller et al., 2005; Simões et al., 2018; Stehman et al., 2007; Tai et al., 2002; Vergnolle and Taylor, 2007). To explore further, we first sought to quantify the dynein-dependent stripping of kinetochore protein cargos. Previous work used an azide-based ATP reduction assay that caused enrichment of cargoes at spindle poles (Howell et al., 2001). This is a rather nonspecific treatment, however, and we reasoned that an alternative approach could be the comparison of cells arrested in nocodazole (where cargoes are stably bound) and monastrol (where cargoes are stripped away from the syntelic (end-on) kinetochore-pairs; see Fig. 4 a). By combining with dynein heavy chain (DHC) siRNA, we can establish the motor’s contribution to corona composition (Fig. 4 b). To validate our “stripping assay,” we compared the intensities of CENP-E, Mad2, and Zwlich at kinetochores in cells treated with siControl + nocodazole or siControl + monastrol and found that levels were reduced to 43.5 ± 35.8%, 22.7 ± 33.7%, and 31.6 ± 18.5%, respectively, in the siControl + monastrol condition (Fig. 4, c–h; *n* ≥ 2, 200 KTs/20 cells per condition). Any expanded crescent-like spots were excluded from the monastrol analysis, as this suggests that the kinetochore is unattached. DHC depletion in monastrol-arrested cells rescued CENP-E, Mad2, and Zwilch localization to 89 ± 78.3%, 58.3 ± 43.8%, and 60.1 ± 28.5%, respectively (Fig. 4, c–h; *n* ≥ 2, 200 KTs/20 cells per condition). We also confirmed that DHC depletion did not dramatically affect microtubule attachments in monastrol-treated cells by quantifying the levels of SKAP, an established marker for mature end-on attachments (Schmidt et al., 2010; Fig. S4, a and b; *n* ≥ 2, 190 KTs/19 cells per condition). Together, these data confirm that (1) the nocodazole/monastrol assay can be used to probe dynein-mediated stripping of kinetochore proteins, and (2) CENP-E, Mad2, and Zwlich are dynein cargoes (Fig. 4, c–h; *n* ≥ 2, 200 KTs/20 cells per condition). We then tested the contribution of CENP-F to cargo transport using our CENP-F-Mut1 and CENP-F-Mut2 cell lines. We found that CENP-E levels were further reduced to 9.6 ± 13.6% and 5.1 ± 7.8% in siControl + monastrol–treated CENP-F-Mut1 and CENP-F-Mut2 cells, respectively. In both cases, CENP-E levels were partially rescued by siDHC treatment (Fig. 5, a and b; *n* = 2, 200 KTs/20 cells per condition). Consistently, we observed a similar dynein-dependent overstripping of Mad2 and Zwlich in the CRISPR cell lines (Fig. 5, c–f; *n* ≥ 2, 180 KTs/18 cells per condition). The partial rescue likely reflects the incomplete depletion of dynein (Fig. 4 b), but nonetheless raises the possibility that CENP-F negatively influences dynein activity to prevent excessive cargo stripping.

**Figure 6.**
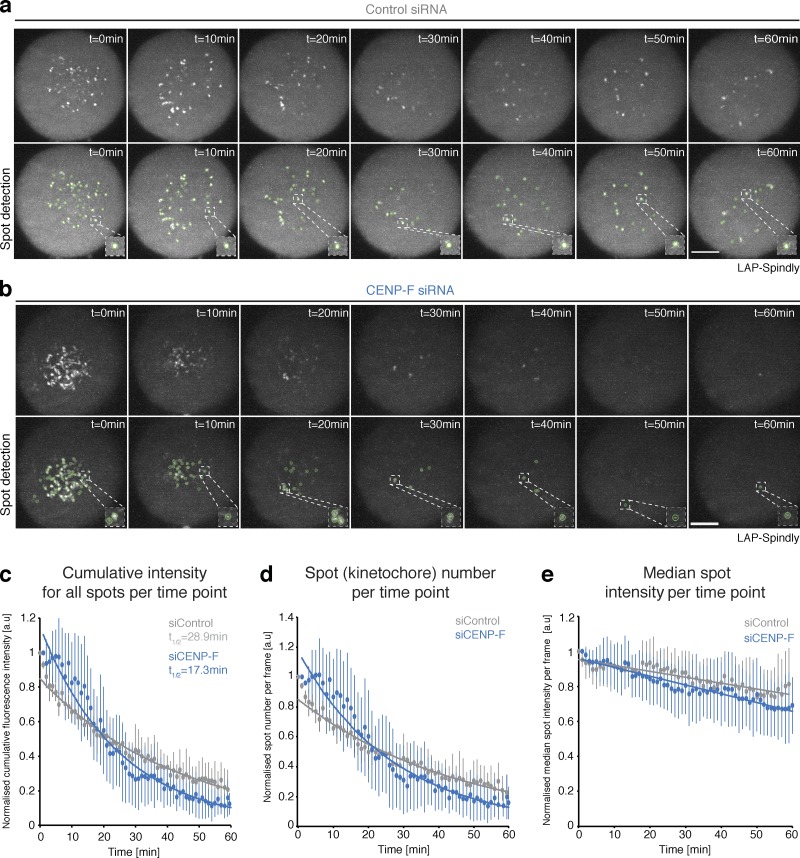
**Lattice light sheet imaging of Spindly dynamics in live cells. (a)** Video stills of siControl-treated HeLa cells expressing doxycycline-inducible LAP-Spindly after nocodazole washout. Cells were arrested in 3.3 µM nocodazole for 4 h and washed once, and a full cell volume was acquired using a lattice light sheet microscope every 1 min for 1 h. Insets show zooms of single kinetochores from the detection panel. Scale bar, 5 µm. **(b)** Video stills of siCENP-F–treated HeLa cells expressing doxycycline-inducible LAP-Spindly after nocodazole washout. Cells were arrested in 3.3 µM nocodazole for 4 h and washed once, and a full cell volume was acquired using a lattice light sheet microscope every 1 min for 1 h. Insets show zooms of single kinetochores from the detection panel. Scale bar, 5 µm. **(c)** Quantification of the cumulative LAP-Spindly intensity for all kinetochores at each time point over the 60-min video for siControl- and siCENP-F–treated cells. Measurements were made using TrackMate in Fiji. Error bars are ±SD. **(d)** Quantification of the spot number at each time point over the 60-min video for siControl- and siCENP-F–treated cells. Measurements were made using TrackMate in Fiji. Errors bars are ±SD. **(e)** Quantification of the median spot intensity at each time point over the 60-min video for siControl- and siCENP-F–treated cells. Measurements were made using TrackMate in Fiji. Errors bars are ±SD.

One limitation of our stripping assay is that it does not allow dynein activity to be followed as end-on attachments form and mature. To address this, we developed a live-cell assay using lattice light sheet microscopy and cell lines stably expressing GFP-tagged Spindly, the adapter molecule for kinetochore dynein and established readout of dynein stripping activity (Gama et al., 2017; Gassmann et al., 2010; Sacristan et al., 2018). GFP-Spindly–expressing cells were treated with siControl or siCENP-F, released from a 4-h nocodazole arrest, and imaged every 1 min for 1 h. This permitted extended imaging of GFP-Spindly as the spindle and kinetochore-microtubule attachments formed from a normalized mitotic configuration. To quantify stripping dynamics, we first plotted the normalized cumulative fluorescence intensity for all spots at each time point. The measurement captures the global Spindly dynamics for all kinetochores as they undergo error correction, biorientation, and congression following nocodazole release. We found that the cumulative GFP-Spindly signal in control cells decreased exponentially, with a *T*_1/2_ of 28.9 min (Fig. 6, a, c, and d; Video 1; *n* = 2, 5 cells per condition). The removal of GFP-Spindly was accelerated in siCENP-F–treated cells, with a *T*_1/2_ of 17.3 min (Fig. 6, b and c; Video 2; *n* = 2, 5 cells per condition). Because the cumulative measurement is a function of both spot number and spot intensity, we plotted these data individually. This revealed that while the spot number followed an almost identical exponential trend to the cumulative data (Fig. 6 d), the median intensity of individual kinetochores decayed by ∼20% in a slow linear manner and was largely unaffected by the loss of CENP-F (Fig. 6 e). This shows that GFP-Spindly localization (at 1-min temporal resolution) is predominantly binary, and CENP-F depletion increases the rate at which kinetochores transition from a “Spindly on” to a “Spindly off” state. Taken together, these data confirm that CENP-F negatively influences the removal of Spindly-dynein from kinetochores as microtubule attachments form.

**Video 1.** **Live-cell visualization of stably integrated and doxycycline-induced GFP-Spindly in siControl-treated HeLa cells.** Cells were arrested in 3.3 µM nocodazole for ∼4 h and washed once, and a full cell volume was acquired every 1 min for 1 h using a lattice light sheet microscope. 7 fps.

**Video 2.** **Live-cell visualization of stably integrated and doxycycline-induced GFP-Spindly in siCENP-F–treated HeLa cells.** Cells were arrested in 3.3 µM nocodazole for ∼4 h and washed once, and a full cell volume was acquired every 1 min for 1 h using a lattice light sheet microscope. 7 fps.

### CENP-F restricts dynein-stripping activity via direct interaction with Nde1

**Figure 7.**
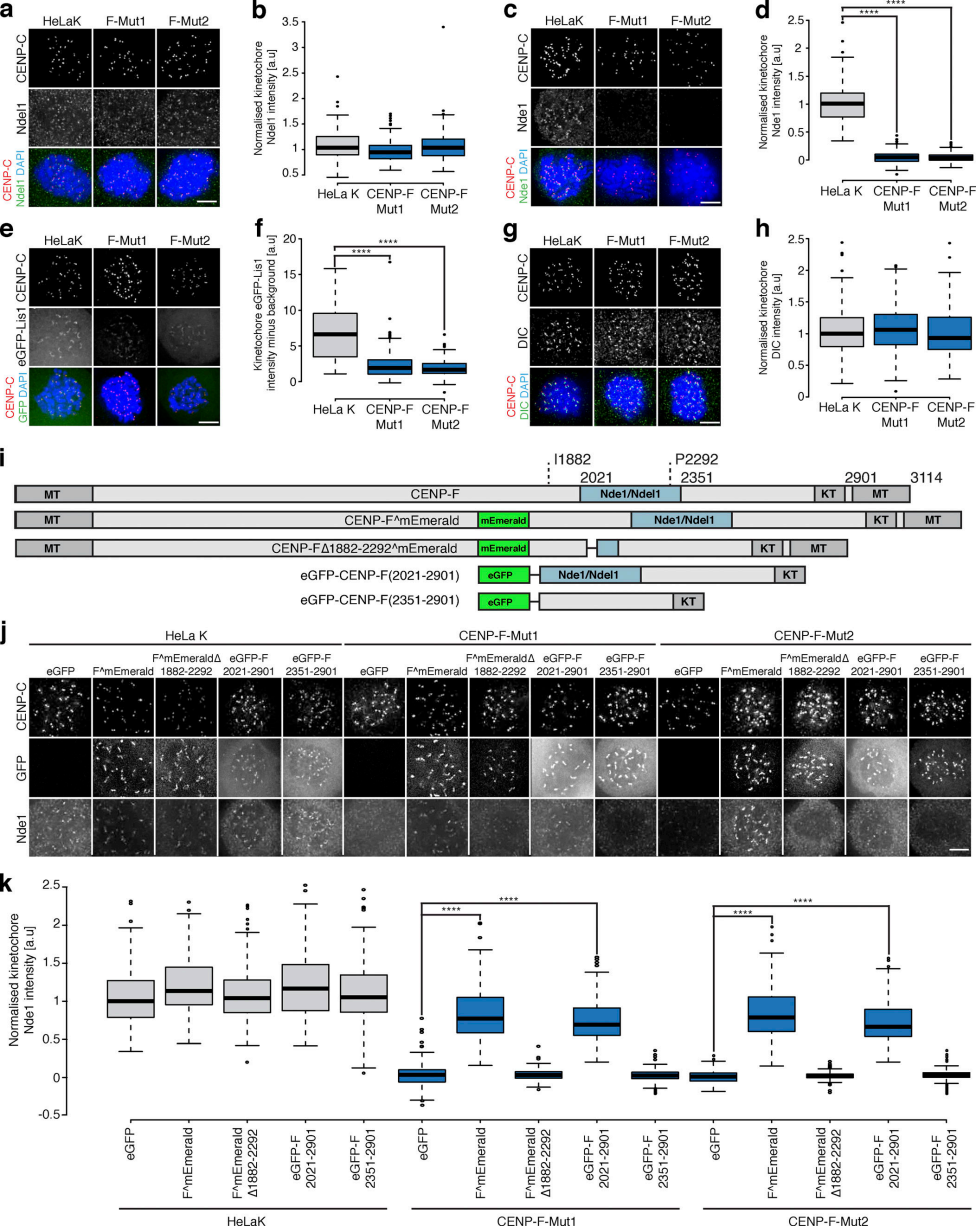
**CENP-F directly controls Nde1 loading to kinetochores. (a)** Immunofluorescence microscopy images of HeLa-K, CENP-F-Mut1, and CENP-F-Mut2 cells arrested in 330 nM nocodazole for 16 h and stained with DAPI and antibodies against CENP-C and Ndel1. Scale bar, 5 µm. **(b)** Quantification of kinetochore Ndel1 levels relative to CENP-C in HeLa-K, CENP-F-Mut1, and CENP-F-Mut2 cells arrested in 330 nM nocodazole for 16 h. **(c)** Immunofluorescence microscopy images of HeLa-K, CENP-F-Mut1, and CENP-F-Mut2 cells arrested in 330 nM nocodazole for 16 h and stained with DAPI and antibodies against CENP-C and Nde1. Scale bar, 5 µm. **(d)** Quantification of kinetochore Nde1 levels relative to CENP-C in HeLa-K, CENP-F-Mut1, and CENP-F-Mut2 cells arrested in 330 nM nocodazole for 16 h. **(e)** Immunofluorescence microscopy images of HeLa-K, CENP-F-Mut1, and CENP-F-Mut2 cells expressing eGFP-Lis1, arrested in 330 nM nocodazole for 16 h, and stained with DAPI and an antibody against CENP-C. Scale bar, 5 µm. **(f)** Quantification of kinetochore eGFP-Lis1 levels after background subtraction in HeLa-K, CENP-F-Mut1, and CENP-F-Mut2 cells arrested in 330 nM nocodazole for 16 h. **(g)** Immunofluorescence microscopy images of HeLa-K, CENP-F-Mut1, and CENP-F-Mut2 cells arrested in 330 nM nocodazole for 16 h and stained with DAPI and antibodies against CENP-C and DIC. Scale bar, 5 µm. **(h)** Quantification of kinetochore DIC levels relative to CENP-C in HeLa-K, CENP-F-Mut1, and CENP-F-Mut2 cells arrested in 330 nM nocodazole for 16 h. **(i)** Cartoon schematic of Nde1-binding mutants. **(j)** Immunofluorescence microscopy images of HeLa-K, CENP-F-Mut1, and CENP-F-Mut2 cells transfected with eGFP, CENP-F^mEmerald, or the Nde1-binding mutants, arrested in 330 nM nocodazole for 16 h, and stained with antibodies against CENP-C and Nde1. Scale bar, 5 µm. **(k)** Quantification of kinetochore Nde1 levels relative to CENP-C in HeLa-K, CENP-F-Mut1, and CENP-F-Mut2 cells transfected with eGFP, CENP-F^mEmerald, or the Nde1-binding mutants and arrested in nocodazole. In b, d, f, h, and k, boxes depict the median and first and third quartiles, and whiskers represent Q1 and Q3 ± 1.5× interquartile range. ****, P < 0.0001.

**Figure 8.**
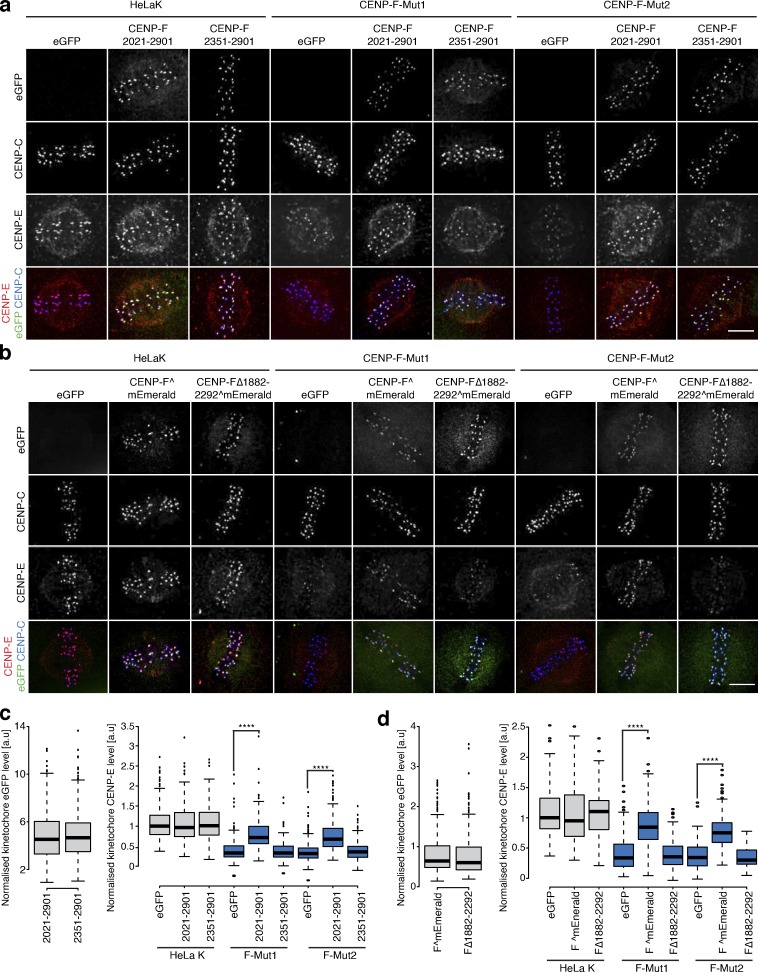
**CENP-F-Nde1 controls corona stripping by dynein in untreated cells. (a)** Immunofluorescence microscopy images of HeLa-K, CENP-F-Mut1, and CENP-F-Mut2 cells transfected with an empty vector, eGFP-CENP-F(2021–2901), or eGFP-CENP-F(2351–2901) and stained with DAPI and antibodies against CENP-E and CENP-C. Scale bar, 5 µm. **(b)** Immunofluorescence microscopy images of HeLa-K, CENP-F-Mut1, and CENP-F-Mut2 cells transfected with an empty vector, CENP-F^mEmerald, or CENP-FΔ1882–2292^mEmerald and stained with DAPI and antibodies against CENP-E and CENP-C. Scale bar, 5 µm. **(c)** Left: Quantification of kinetochore eGFP intensities minus background in cells expressing eGFP-CENP-F(2021–2901) or eGFP-CENP-F(2351–2901). Right: Quantification of kinetochore CENP-E intensities relative to CENP-C in HeLa-K, CENP-F-Mut1, and CENP-F-Mut2 cells transfected with an empty vector, eGFP-CENP-F(2021–2901), or eGFP-CENP-F(2351–2901). **(d)** Left: Quantification of kinetochore eGFP intensities minus background in cells expressing CENP-F^mEmerald or CENP-FΔ1882–2292^mEmerald. Right: Quantification of kinetochore CENP-E intensities relative to CENP-C in HeLa-K, CENP-F-Mut1, and CENP-F-Mut2 cells transfected with an empty vector, CENP-F^mEmerald, or CENP-FΔ1882–2292^mEmerald. In c and d, boxes depict the median and first and third quartiles, and whiskers represent Q1 and Q3 ± 1.5× interquartile range. ****, P < 0.0001.

**Figure 9.**
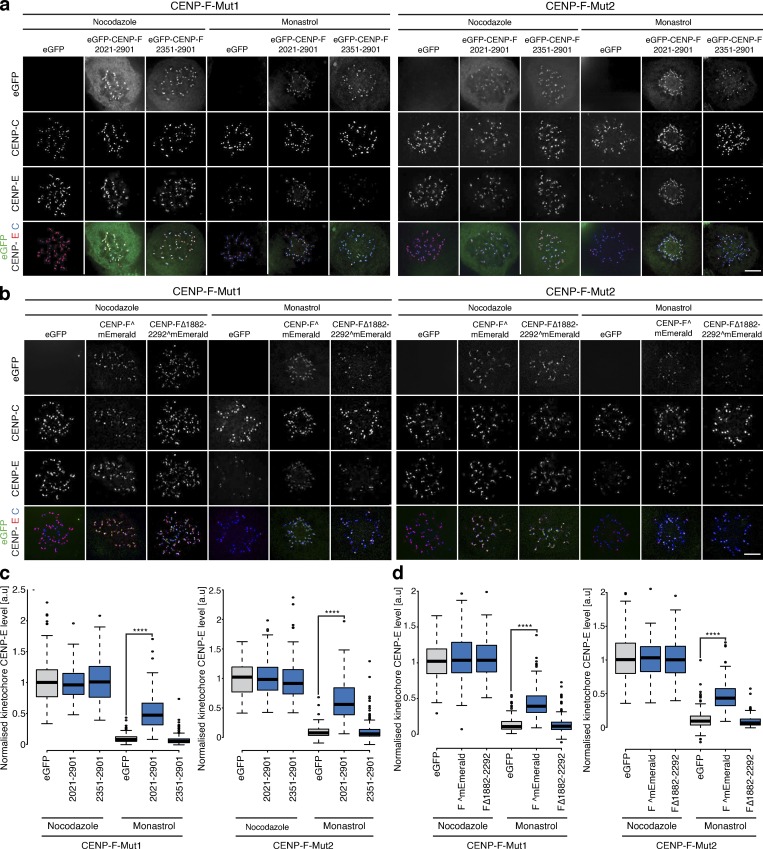
**CENP-F-Nde1 controls corona stripping by dynein the nocodazole-monastrol assay. (a)** Immunofluorescence microscopy images of the nocodazole-monastrol dynein-stripping assay in CENP-F-Mut1 and CENP-F-Mut2 cells transfected with an empty vector, eGFP-CENP-F(2021–2901), or eGFP-CENP-F(2351–2901) and stained with antibodies against CENP-E and CENP-C. Scale bar, 5 µm. **(b)** Immunofluorescence of the nocodazole-monastrol dynein-stripping assay in CENP-F-Mut1 and CENP-F-Mut2 cells transfected with an empty vector, CENP-F^mEmerald, or CENP-FΔ1882–2292^mEmerald and stained with antibodies against CENP-E and CENP-C. Scale bar, 5 µm. **(c)** Quantification of kinetochore CENP-E intensities relative to CENP-C in the nocodazole-monastrol assay depicted in a. **(d)** Quantification of kinetochore CENP-E intensities relative to CENP-C in the nocodazole-monastrol assay depicted in b. In c and d, boxes depict the median and first and third quartiles, and whiskers represent Q1 and Q3 ± 1.5× interquartile range. ****, P < 0.0001.

How does CENP-F influence dynein motor behavior? A key candidate is the Nde1-Ndel1-Lis1 complex that physically contacts both CENP-F and dynein (Olenick and Holzbaur, 2019; Vergnolle and Taylor, 2007). We thus quantified the localization of Ndel1, Nde1, eGFP-Lis1, and dynein intermediate chain (DIC) at kinetochores in CENP-F-Mut1 and CENP-F-Mut2 cells. This revealed that while Nde1 and Lis1 levels were reduced (to 4.8 ± 9.7% and 4.6 ± 7.6% for Nde1 and 34.2 ± 28.3% and 28.1 ± 16.5% for Lis1), the loss of CENP-F had no effect on the kinetochore intensities of Ndel1 and DIC (Fig. 7, a–h; *n* = 2, 200 KTs/20 cells per condition). Presumably, dynein is tethered at kinetochores via redundant interactions, such as with Spindly-RZZ (Gama et al., 2017; Gassmann et al., 2010). We note that this result differs from earlier work in which both Ndel1 and dynein were absent from kinetochores in siCENP-F–treated cells (Vergnolle and Taylor, 2007). To further the functional characterization of CENP-F-Nde1, we created a full-length CENP-F containing a deletion between I1882 and P2292, which maintains the predicted C-terminal coiled-coil structure while removing most of the Nde1-binding domain (CENP-FΔ1882–2292^mEmerald; Figs. 7 i and S4, e and f). Unlike the full-length CENP-F^mEmerald, transfection of CENP-F-Mut1 and Mut2 cells with the Nde1-binding domain deletion failed to rescue Nde1 binding to kinetochores (Fig. 7, j and k; *n* = 2, 200 KTs/20 cells per condition). To test whether the Nde1-binding domain can target Nde1 to kinetochores, we transfected cells with a transgene that contains the Nde1-binding domain, an adjacent coiled-coil region and the KTD (eGFP-CENP-F(2021–2901); Stehman et al., 2007; Vergnolle and Taylor, 2007; Wynne and Vallee, 2018; Zhu et al., 1995; Fig. 7 i). This minimal CENP-F was sufficient for Ndel1 recruitment (Fig. 7, j and k; *n* = 2 200 KTs/20 cells per condition). As a control, we confirmed that the same transgene in which the Nde1-binding domain is removed, leaving only the coiled-coil region and KTD (eGFP-CENP-F(2351–2901) abolished the rescue (Fig. 7, j and k; *n* = 2, 200 KTs/20 cells per condition). Thus, the Nde1-binding domain is necessary for Nde1 loading to kinetochores. Expression of the CENP-F variants that restored Nde1 localization also rescued the localization of CENP-E motors onto metaphase kinetochores in CENP-F-Mut1 and Mut2 cells (Fig. 8, a–d; *n* ≥ 2, 200 KTs/20 cells per condition). In contrast, the CENP-FΔ1882–2292^mEmerald and eGFP-CENP-F(2351–2901) constructs that failed to support Nde1 binding also failed to rescue CENP-E localization (Fig. 8, a–d; *n* ≥ 2, 200 KTs/20 cells per condition). This is consistent with the CENP-F–Nde1 interaction being essential for restricting dynein-dependent stripping. To substantiate this idea, we combined the nocodazole-monastrol assay (Fig. 4 a) with expression of these constructs in CENP-F-Mut1 and CENP-F-Mut2 cells. Consistent with our previous data, we found that while CENP-F^mEmerald and eGFP-CENP-F(2021–2901) could partially restore CENP-E localization in monastrol-treated cells, expression of CENP-FΔ1882–2292^mEmerald or eGFP-CENP-F(2351–2901) failed to prevent CENP-E overstripping (Fig. 9, a–d; *n* = 2, 200 KTs/20 cells per condition). We note that eGFP-CENP-F(2021–2901) does not contain the proposed CENP-E–binding site (Chan et al., 1998), confirming that a direct interaction between CENP-F and the motor is not necessary for kinetochore localization. Taken together, these data demonstrate that CENP-F restricts dynein-stripping activity through a physical interaction with Nde1.

### CENP-F-Nde1 module is required for error correction

Our data reveal how CENP-F has two functional modules, one that provides a direct binding interface for microtubules and one that influences dynein stripping of kinetochore cargoes. How are these activities integrated to ensure accurate and timely chromosome segregation? We have already established that CENP-F MTBD mutants reduce K-K tension, suggesting that there are lower forces being exerted by microtubule–kinetochore interactions.

**Figure S5.**
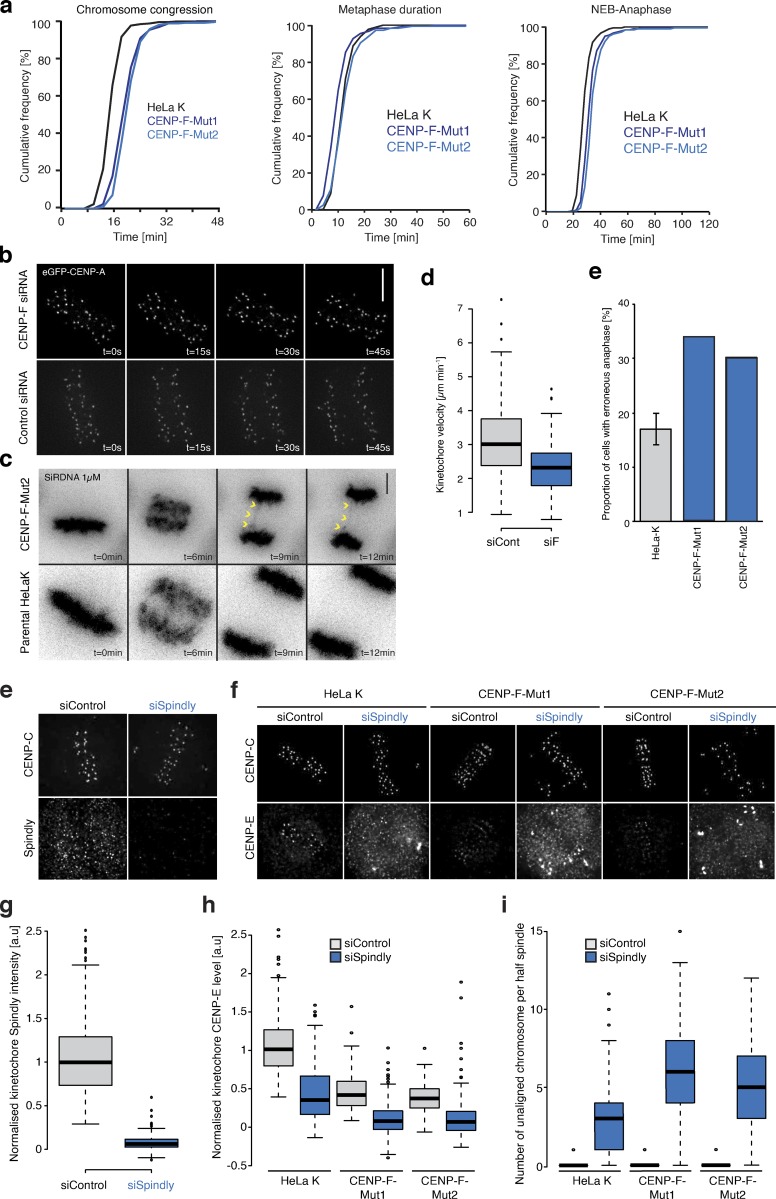
**Loss of CENP-F perturbs mitotic progression and influences the Spindly dependent retention of CENP-E.** **(a)** Cumulative frequency plots of congression time, metaphase duration, and nuclear envelope breakdown-anaphase in HeLa-K, CENP-F-Mut1, and CENP-F-Mut2 cells visualized with 1 µM SiRDNA. **(b)** Video stills of anaphase eGFP-CENP-A–expressing cells treated with either control or CENP-F siRNA. Scale bar, 5 µm. **(c)** Images of HeLa-K or CENP-F-Mut2 cells labeled with 1 µM SiRDNA progressing though anaphase. Yellow arrows indicate an error. Scale bar, 5 µm. **(d)** Quantification of anaphase kinetochore velocity in eGFP-CENP-A–expressing HeLa cells treated with control or CENP-F siRNA. Velocity measurements were taken from tracks of processive movement that lasted at least three time frames. **(e)** Quantification of anaphase error rates in HeLa-K, CENP-F-Mut1, and CENP-F-Mut2 cells. **(f)** Immunofluorescence microscopy images of HeLa-K cells treated with control or Spindly siRNA and stained with DAPI and antibodies against CENP-C and Spindly. Scale bar, 5 µm. **(g)** Quantification of kinetochore Spindly level relative to CENP-C in HeLa-K cells treated with control or Spindly siRNA. **(h)** Immunofluorescence microscopy images of HeLa-K, CENP-F-Mut1, and CENP-F-Mut2 cells treated with control or Spindly siRNA and stained with DAPI and antibodies against CENP-C and CENP-E. Scale bar, 5 µm. **(i)** Quantification of kinetochore CENP-E level relative to CENP-C in HeLa-K, CENP-F-Mut1, and CENP-F-Mut2 cells treated with control or Spindly siRNA. **(j)** Quantification of the number of unaligned chromosomes per half-spindle in HeLa-K, CENP-F-Mut1, and CENP-F-Mut2 cells treated with control or Spindly siRNA. In d, g, h, and i, boxes depict the median and first and third quartiles, and whiskers represent Q1 and Q3 ± 1.5× interquartile range.

Nevertheless, we found by long-term time-lapse imaging of CENP-F CRISPR mutant cell lines (and also CENP-F RNAi–treated cells; not depicted) that these defects in kinetochore function had limited effect on mitotic progression: cells transited though mitosis and initiated anaphase with only a mild ∼6-min delay in chromosome congression (10% unaligned at *T* = 24 min in CENP-F-Mut1 and CENP-F-Mut2 cells compared with 1% in control cells; Fig. S5 a; *n* ≥ 3, 300 cells). Anaphase was largely normal, although we could detect a minor increase in anaphase errors (Fig. S5, c and e; *n* ≥ 3, 300 cells) and reduced kinetochore poleward velocities (3.14 ± 1.2 µm min^−1^ in controls cells to 2.34 ± 0.8 µm min^−1^ in CENP-F–depleted cells, Fig. S5, b and d; *n* ≥ 2, 100 KTs/15 cells per condition).

**Figure 10.**
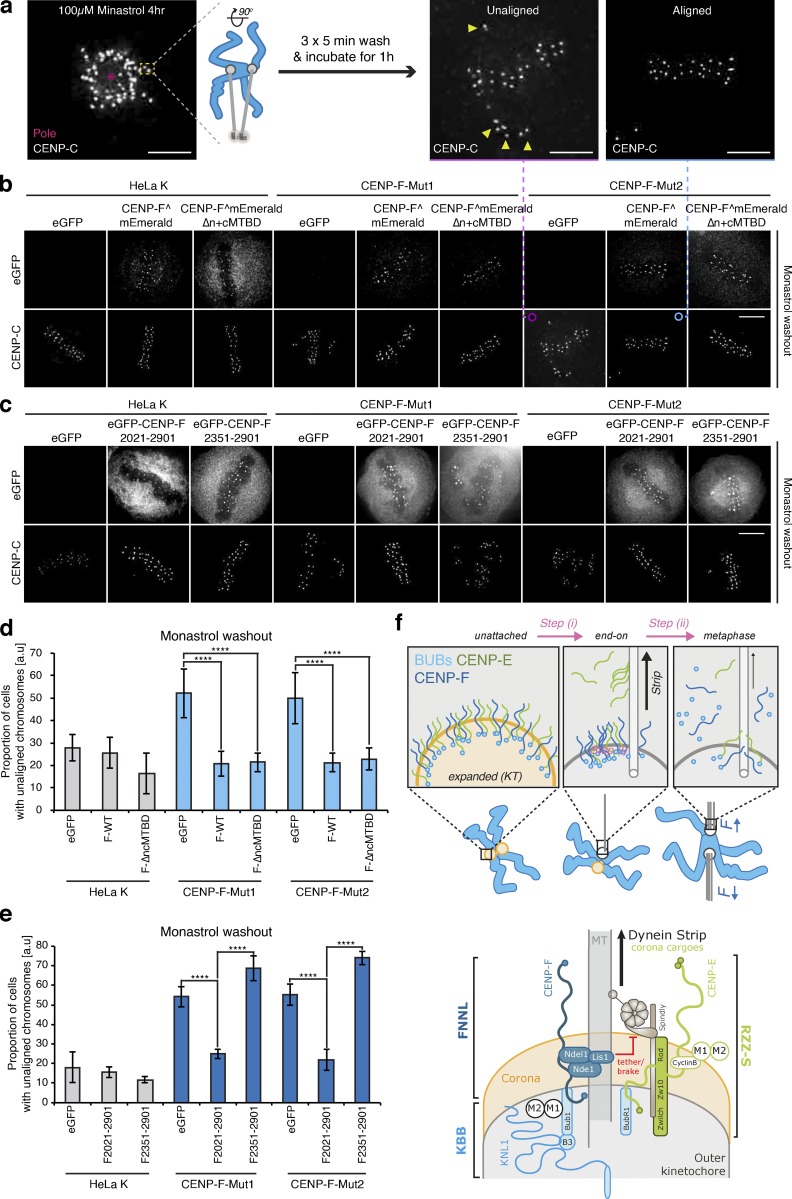
**Distinct functional contributions of Nde1 and MT binding by CENP-F. (a)** Schematic depicting the monastrol release experiment. Scale bar, 5 µm. **(b)** Immunofluorescence microscopy images of HeLa-K, CENP-F-Mut1, and CENP-F-Mut2 cells transfected with eGFP, CENP-F^mEmerald, or CENP-FΔn+cMTBD^mEmerald, released from a monastrol block for 1 h, and stained with an antibody against CENP-C. Scale bar, 5 µm. **(c)** Immunofluorescence microscopy images of HeLa-K, CENP-F-Mut1, and CENP-F-Mut2 cells transfected with eGFP, eGFP-CENP-F(2021–2901), or eGFP-CENP-F(2351–2901), released from a monastrol block for 1 h, and stained with an antibody against CENP-C. Scale bar, 5 µm. **(d)** The proportion of HeLa-K, CENP-F-Mut1, and CENP-F-Mut2 cells transfected with eGFP, CENP-F^mEmerald, or CENP-FΔn+cMTBD^mEmerald with unaligned chromosomes 1 h after release from monastrol block. **(e)** The proportion of HeLa-K, CENP-F-Mut1, and CENP-F-Mut2 cells transfected with eGFP, eGFP-CENP-F(2021–2901), or eGFP-CENP-F(2351–2901) with unaligned chromosomes 1 h after release from monastrol block. **(f)** Top: A working model of coronal stripping as end-on attachments form and mature: as end-on attachments form (step i), Spindly-bound dynein motor complexes are released and begin striping cargoes (including CENP-E, Mad2, and Zwlich) from the kinetochore. As these sister-pairs biorient and align in metaphase (step ii), Bub1 is also lost, leading to a reduction in CENP-F and further dynein stripping. Bottom: Schematic depicting molecular basis for dynein recruitment and regulation at the kinetochore. Spindly-bound dynein is released after end-on attachment to strip a myriad of coronal cargoes (green). RZZ binds directly to Spindly, while the physical interactions that bridge CENP-E (which binds BubR1) and dynein motors is unknown. Mad2–Mad1 complexes bind Cyclin B in the corona, although how this bridges to dynein is also unknown. The CENP-F dynein brake/tether, which includes Nde1 and potentially Lis1, is loaded to kinetochores via the direct interaction of CENP-F with Bub1, a component of the KNL3-Bub3-Bub1 (KBB) pathway (light blue). CENP-F-Nde1-Ndel1-Lis1 (FNNL; dark blue) limits the strip rate to ensure kinetochores have the correct protein stoichiometry in the corona. In d and e, boxes depict the median and first and third quartiles, and whiskers represent Q1 and Q3 ± 1.5× interquartile range. ****, P < 0.0001.

This hints that there are certain physiological circumstances in which the CENP-F functional modules are required. To explore this idea further, we treated cells with the kinesin-5 inhibitor monastrol, which traps the majority of kinetochores in a syntelic state around a monopole. By releasing cells from the block, all kinetochores must now undergo error correction, lateral-to-end-on conversion, biorientation, and finally congression to the spindle equator (Fig. 10 a). The fraction of cells with unaligned kinetochores after 1 h thus serves as a readout of kinetochore function. Because this is an end-point assay using fixed-cell imaging, we were also able to phenotype cells that had wild-type or mutant CENP-F transgenes localized to kinetochores. The fraction of cells with unaligned kinetochores was elevated in both CENP-F-Mut1 and CENP-Mut2 cells (with ∼60% of CENP-F-Mut1 and CENP-F-Mut2 cells with unaligned chromosomes vs. ∼20% in control cells; Fig. 10, b–e; *n* ≥ 3, 100 cells per condition). This phenotype could be rescued by transfection of the wild-type CENP-F transgene and with a CENP-F construct that lacks both MTBDs (Fig. 10, b and d; *n* ≥ 3, 100 cells per condition). This result suggests that the loss of K-K tension and attachment stability following loss of the MTBDs does not impact the error correction and biorientation of kinetochores. This result also suggests that these processes are affected by the overstripping of dynein cargos. To test this, we transfected HeLa-K, CENP-F-Mut1, and CENP-F-Mut2 cells with eGFP-CENP-F(2021–2901), which contains only the KTD and Nde1-binding domains. This CENP-F truncation alone was able to rescue the chromosome congression phenotype (∼25% of CENP-F-Mut1 and CENP-F-Mut2 cells with unaligned chromosomes compared with ∼19% in control cells after 1-h release from monastrol block), while a control construct that lacks the Nde1-binding domain (eGFP-CENP-F(2351–2901)) was not able to rescue the phenotype (Fig. 10, c and e; and Fig. S4 f; *n* ≥ 3, 100 cells per condition). Taken together, these data reveal how CENP-F modulation of dynein cargos (including CENP-E and Zwlich) is crucial for efficient conversion of syntelic attachments into aligned and bioriented sister kinetochores.

## Discussion

In this study, we have defined for the first time how the two CENP-F MTBDs contribute to kinetochore activity in human cells: both MTBDs are required for the generation of normal tension across centromeres. It is important to note that the K-K distance is not reduced to rest length, indicating that some force is still being exerted through the kinetochore. Indeed, in cells lacking CENP-F, kinetochores can still align on the metaphase plate and sister chromatids can be segregated, although the semiperiodic oscillations at metaphase and poleward motion in anaphase are slowed and the error rate is increased. This substantiates the idea that lowered centromere tension does not interfere with checkpoint satisfaction but may affect the correction of merotelic attachments in anaphase (Dudka et al., 2018). Loss of the amino-terminal MTBD also reduced the cold stability of kinetochore-microtubule attachments without affecting K-fiber formation. The phenotypic differences between the amino-/carboxy-terminal MTBDs may reflect subtle differences in their biochemical properties, as the amino-terminal MTBD preferentially binds curved microtubule structures, while the carboxy-terminal MTBD has a higher affinity for straight lattice configurations (Volkov et al., 2015). Nevertheless, our data are consistent with in vitro biophysical experiments that demonstrate how CENP-F can function as a force transducer (Kanfer et al., 2017; Volkov et al., 2015). This is, however, distinct from the Ska complex, which has a well-established force-transducing role in vitro and during congression in vivo (Auckland et al., 2017; Helgeson et al., 2018; Huis In ’t Veld et al., 2019b). We did not find a substantial role for CENP-F in congression and also found no additive defects when the Ska complex was depleted in CENP-F mutant cells (not depicted), thus ruling out any redundancy. Finally, our phenotypes in RNAi and CRISPR cell lines are consistent with recent reports that CENP-F is a nonessential gene in human (McKinley and Cheeseman, 2017; Raaijmakers et al., 2018) and mouse (Haley et al., 2019) cells.

Our data also show how both MTBDs are dispensable for the efficient correction and biorientation of erroneous microtubule-kinetochore attachments generated following a monastrol washout. Instead, we have established that it is the Nde1-binding domain of CENP-F that is crucial for these processes. Our data reveal how the Nde1–CENP-F interaction influences the rate of corona contraction and disassembly by limiting the removal of cargos (including CENP-E, Zwilch, Spindly, and Mad2) by dynein motors from kinetochores. This builds on the earlier observation that CENP-F can protect CENP-E, dynein, and dynactin from microtubule-dependent stripping (Yang et al., 2005). Whether kinetochore cargos are removed by distinct dynein-dependent stripping events or as a single larger complex, such as the previously described RZZ-MES supercomplex (containing Rod, Zwilch, ZW10, Mad1, CENP-E, and Spindly; Samejima et al., 2015), is unclear. We currently favor the former, as while some cargoes are partially removed from kinetochores (i.e., CENP-E, Zwlich) others are completely stripped (i.e., Mad2, Spindly). Indeed, our live imaging showed that kinetochores switch from a Spindly(+) to Spindly(−) state on a timescale of minutes. This is consistent with measurements of Mad1 loss during the formation of end-on attachments (*T*_1/2_ = ∼79 s; Kuhn and Dumont, 2017). We note that there is also evidence for a slow loss of Spindly from unattached/laterally attached kinetochores, although the mechanism involved is unknown. Nevertheless, the kinetics of all cargoes will need to be understood to build a complete picture of the stripping system.

Our working model (Fig. 10 f, upper panel) is that as end-on attachments form (step i), dynein motors are released and begin stripping cargoes (including CENP-E, Mad2, and Zwlich) from the kinetochore. The CENP-F-Nde1 tether/brake puts a limit on dynein stripping to ensure that a pool of coronal components remain on kinetochores. As sister kinetochores biorient and align in metaphase (step ii), Bub1 is also lost from kinetochores as the checkpoint is satisfied and attachments mature (Auckland et al., 2017; (Etemad et al., 2019)). This would contribute to a reduction in dynein stripping through loss of CENP-F from kinetochores. There are two possible mechanisms that can explain how the CENP-F–Nde1 interaction can influence dynein motor behavior on kinetochores: (1) Bub1-bound CENP-F-Nde1 may act as a “dynein tether,” restricting the rate of dynein release from kinetochores through the physical interaction of Nde1 with the motor (Fig. 10 f, lower panel); or (2) CENP-F-Nde1 could operate as a “dynein brake” to slow/inhibit dynein motor biochemistry at kinetochores. In line with this, both our work and that of others (Vergnolle and Taylor, 2007) have found that CENP-F-Nde1 is required to load a large pool of Lis1 to kinetochores. Lis1 is a context-dependent dynein regulator that has been shown to both positively and negatively regulate dynein at distinct subcellular locations in several model systems (Baumbach et al., 2017; DeSantis et al., 2017; Dix et al., 2013; Faulkner et al., 2000; Gutierrez et al., 2017; Huang et al., 2012; Klinman and Holzbaur, 2015; Moughamian et al., 2013; Pandey and Smith, 2011; Shao et al., 2013; Siller et al., 2005; Simões et al., 2018; Toropova et al., 2014; Vagnoni et al., 2016; Yi et al., 2011). In vitro reconstitution experiments using dynein-Spindly will be required to determine the function of Lis1 in a kinetochore context. One prediction from our data is that depletion of Nde1 itself should also lead to premature stripping of dynein cargos from kinetochores. However, it has been shown that dynein unbinds from kinetochores following depletion of Nde1 (Vergnolle and Taylor, 2007; Wynne and Vallee, 2018). Thus, even when not bound to CENP-F, Nde1 retains some capacity to tether or stabilize dynein motor complexes at kinetochores. It will also be important to understand how the CENP-F–Nde1 dynein-brake/tether is coordinated with RZZ-S, which recruits dynein to kinetochores (Gama et al., 2017). Our initial experiments show that depletion of Spindly in CENP-F mutant cells completely abolishes CENP-E binding at metaphase kinetochores (Fig. S5, e–i). This supports the idea that both Spindly and CENP-F tether dynein motors at kinetochores. But since Spindly is needed for dynein activity, it also suggests that other, as yet unknown, mechanisms can trigger the disassembly of the corona.

Because the dynein brake/tether activity is sufficient to rescue the error correction/biorientation defects following monastrol washout, we propose that CENP-F–Nde1 plays a crucial role in ensuring that kinetochores retain the optimal number of corona components to enable efficient transition between key attachment states (i.e., syntelic → unattached → lateral → end-on). For example, it has been shown how reducing the number of CENP-E motors on kinetochores perturbs the efficient conversion from lateral to end-on attachment (Shrestha and Draviam, 2013). However, at this stage, we cannot rule out the possibility that CENP-F–Nde1 regulation of dynein also impacts the motor’s direct role in attachment and biorientation independently of cargo stripping. While CENP-F-Nde1 governs the rate of corona disassembly, we found that CENP-F is not required for assembly of the corona at unattached kinetochores. Expansion of CENP-F within the corona might potentially involve templating on the underlying RZZ-S self-assembling meshwork (Pereira et al., 2018; Sacristan et al., 2018), although the mechanism remains to be determined. Our data do provide a hint that the central region of CENP-F is positioned outside of the carboxy-terminus that contains both microtubule and KTD (see Fig. 3 n). Further work is needed to establish if CENP-F can span the corona-to-outer-kinetochore junction and how expansion takes place in this context.

Overall, our experiments are beginning to shed light on how the kinetochore-bound CENP-F–Nde1-Ndel1–Lis1–dynein axis contributes to early mitotic events in human cells. Beyond this, CENP-F is also located at the outer nuclear envelope and mitochondria, where it functions in centrosome tethering and mitochondrial trafficking, respectively (Berto and Doye, 2018; Berto et al., 2018; Bolhy et al., 2011; Kanfer et al., 2015; Kanfer et al., 2017; Peterka and Kornmann, 2019). It will be essential to determine whether the mechanisms revealed in this study are relevant in these different cellular contexts. This is of particular interest in regard to the localization of CENP-F to the base of cilia and how mutations are associated with the development of human ciliopathies, including Strømme syndrome (Filges, 2016).

## Materials and methods

### Cell culture, siRNA transfection, and drug treatments

HeLa-Kyoto (K), CENP-F-Mut1, CENP-F-Mut2, and HeLa-LAP-Spindly cells were grown in a humidified incubator at 37°C and 5% CO_2_ in DMEM (Gibco) containing 10% FCS (Sigma-Aldrich), 100 U/ml penicillin, and 100 µg/ml streptomycin (Gibco). This was supplemented with 0.1 µg/ml puromycin (Invitrogen) for the maintenance of the eGFP-CENP-A cell line. The hTERT-RPE1-CENP-F(KTD) cell line was maintained in DMEM/F-12 medium containing 10% FCS, 2.3 g/liter sodium bicarbonate, 100 U/ml penicillin, and 100 µg/ml streptomycin. siRNA oligonucleotides (53 nM) were transfected using oligofectamine (Invitrogen) according to the manufacturer’s guidelines and analyzed at 48 h. The following sequences were used: control, 5′-GGA​CCU​GGA​GGU​CUG​CUG​U-3′; CENP-F, 5′-AAG​AGA​AGA​CCC​CAA​GUC​AUC-3′ (Holt et al., 2005); and DHC, 5′-GGA​UCA​AAC​AUG​ACG​GAA​U-3′. For drug treatments, cells were treated with 330 nM nocodazole for 4 or 16 h (see text) or 100 µM monastrol for 4 h before fixation. For monastrol release, cells were arrested for 4 h, washed three times in warm DMEM, and incubated for 1 h before fixation. For overnight imaging, cells were incubated with 1 µM SiRDNA (Spirochrome) for 30 min before imaging. For LAP-Spindly induction, cells were incubated in 1 µM doxycycline (Sigma-Aldrich) for 24 h before imaging.

### Plasmid construction and siRNA rescue experiments

We note that Uniprot and Ensembl previously reported conflicting CENP-F lengths of 3,200 and 3,114 amino acids, respectively (this has now been corrected to 3114). This resulted from a 96-amino acid insertion at position 1515 between two major coiled-coil regions. In the present study, all C-terminal domain positions have been adjusted for this insertion. To generate an siRNA protected version of CENP-F^GFP for transient expression in cells, full-length CENP-F^mEmerald was excised from pcDNA5/frt/to using BamHI and NotI sites and cloned into pcDNA3.1+, creating pcDNA3.1+CENP-F^mEmerald (pMC619). A short DNA fragment containing the siRNA protected sequence (5′-GAA​AAA​ACG​CCT​AGC​CAC​C-3′) was synthesized with flanking BamHI and AgeI sites (pMC618, GeneArt) and cloned into pcDNA3.1+CENP-F^mEmerald to create pcDNA3.1+CENP-F^mEmerald-RIP (pMC621). The pcDNA3.1+CENP-F^mEmerald-RIP plasmid was then used to create all microtubule-binding mutants. For deletion of the N-terminal MTBD, a fragment of CENP-F from the terminus of the MTBD to an internal AgeI site was amplified by PCR with a flanking 5′ BamHI site. This was cloned into pcDNA3.1+CENP-F^mEmerald-RIP using BamHI and AgeI sites, creating pcDNA3.1+CENP-F^mEmeraldΔnMTBD (pMC620). For deletion of the C-terminal MTBD, a fragment of CENP-F from an internal SfiI site to the start of the MTBD was amplified by PCR with a flanking 3′ NotI site. This was cloned into pcDNA3.1+CENP-F^GFP-RIP using SfiI and NotI sites to create pcDNA3.1+CENP-F^mEmerald-RIPΔcMTBD (pMC626). To generate pcDNA3.1+CENP-F^mEmeraldΔn+cMTBD (pMC627), the above cloning was performed sequentially. For siRNA rescue experiments, HeLa K cells were grown on 22-mm 1.5 coverglass for 24 h before treatment with either control or CENP-F siRNA for 24 h in 1.5 ml MEM. Cells were then transfected with 2 µg of Maxiprep plasmid DNA using FugeneHD at 1:4 according to the manufacturer’s guidelines and incubated for a further 48 h in 1.5 ml DMEM. To generate the Nde1/Ndel1 binding mutants, either CENP-F(2021–2901) or CENP-F(2351–2901) was amplified by PCR with flanking Kpn1 and BamHI sites and cloned into peGFP-C1, creating eGFP-CENP-F(2021–2901) (pMC703) and eGFP-CENP-F(2351–2901) (pMC704), respectively. To create CENP-FΔ1882–2292^mEmerald (pMC702), two fragments were amplified by PCR from CENP-F^mEmerald to remove amino acids 1882–2292. These were sequentially cloned into pcDNA3.1+ using BamHI-NotI and NotI sites, respectively.

To generate eGFP-Lis1, full length Lis1 was ordered from Geneart (Thermo Fisher Scientific) and cloned into peGFP-C1 using SalI and MfeI sites to create pMC701. For expression in cells, 2 µg of plasmid DNA was transfected using FugeneHD at 1:4 and incubated for 48 h in 1.5 ml DMEM.

### CRISPR-Cas9

To target CENP-F exons 2 and 19, the guides 5′-CCG​AGG​GTA​CAA​ACC​TGA​AA-3′ (exon2) and 5′-CAG​CGG​AGC​CCA​GTA​GAT​TC-3′ (exon19; Raaijmakers et al., 2018) were cloned into the human codon optimized SpCas9 and chimeric guide expression plasmid (pX330, Addgene) using BbsI as previously described (Ran et al., 2013). To generate CENP-F-Mut1 and CENP-F-Mut2 cell lines, HeLa-K cells grown in a 33-mm dish were simultaneously transfected with 1 µg of each Cas9-guide plasmid using FugeneHD at 1:4 according to the manufacturer’s guidelines and incubated for 48 h in 1.5 ml DMEM. Cells were diluted 10-fold and plated on 15-cm dishes in DMEM without selection and grown for ∼2 wk. Single colonies were picked using trypsin-soaked cloning disks (Sigma-Aldrich), amplified, and screened by immunofluorescence. The exon2 and exon19 alleles in CENP-F mutant clones were amplified by PCR with flanking KpnI and AgeI sites and cloned into peGFP-N1 for sequencing. A summary of the sequencing data can be found in Fig. S2 d. At least 10 alleles were sequenced per cut site.

### Immunofluorescence microscopy

Cells were fixed in 20 mM Pipes, pH 6.8, 10 mM EGTA, 1 mM MgCl_2_, 0.2% Triton X-100, and 4% formaldehyde. For the cold-stable assay, cells were incubated in ice-cold DMEM for 10 min before fixation. Cells were washed three times in PBS before blocking in PBS + 3% BSA for 30 min and incubation with primary antibodies for 1 h at room temperature: anti-CENP-C (1/2,000, guinea pig, MBL), anti-CENP-E (1/1,000, rabbit, gift from P. Meraldi, University of Geneva, Geneva, Switzerland), anti-CENP-F(Ab5) (1/400, rabbit, Abcam), anti-CENP-F(Ab90) (1/400, mouse, Abcam), anti-CENP-A (1/500, mouse, Abcam), anti-α-tubulin (1/1,000, mouse, Sigma-Aldrich), anti-SKAP (1/400, rabbit, Atlas Antibodies), anti-Bub1 (Ab54893, 1/200, mouse, Abcam), CREST antisera (1/250, human, Antibodies Inc.), anti-Mad2 (1/1,000, mouse, Santa Cruz), anti-Zwlich (1/1,000, rabbit, gift from A. Musacchio, Max Planck Institute of Molecular Physiology, Dortmund, Germany), anti-Nde1 (1/500, rabbit, Proteintech), and anti-NdelI (1/500, rabbit, gift from S. Taylor, University of Manchester, Manchester, UK). For anti-DIC (mouse, 1/200, EMD Millipore), cells were fixed in −20°C methanol for 10 min. After fixation, cells were washed three times with PBS and incubated with Alexa Fluor–conjugated secondary antibodies at 1/500 for 1 h at room temperature. Cells were mounted and imaged in Vectashield (Vector Laboratories). For K-fiber analysis, cells were preextracted for 30 s in 80 mM Pipes, pH 6.8, 1 mM MgCl_2_, 4 mM EGTA, and 0.5% Triton X-100 and fixed by adding glutaraldehyde to 0.5% for 10 min. Glutaraldehyde was quenched using a 7-min treatment with 0.1% NaBH_4_. Cells were washed three times in PBS for 5 min, followed by blocking in TBS containing 0.1% Triton X-100 and 2% BSA for 10 min. Cells were then incubated with anti–α-tubulin (1/1,000, mouse, Sigma-Aldrich) primary antibody for 30 min, followed by four 5-min washes in TBS containing 0.1% Triton X-100 and incubation with 647-nm Alexa Fluor–conjugated secondary antibodies (Invitrogen) in TBS containing 0.1% Triton X-100 and 2% BSA for 30 min. Cells were mounted and imaged in Vectashield. 3D image stacks were acquired using a 100× oil NA 1.4 objective on an Olympus Deltavision Elite microscope (Applied Precision) equipped with a DAPI, FITC, Rhodamine, or Texas Red and Cy5 filter set, solid-state light source, and a CoolSNAP HQ2 camera (Roper Technologies) at 37°C. Stacks were deconvolved in SoftWorx (Applied Precision). Fluorescence intensity measurements were taken in SoftWorx using a 5 × 5–pixel region of interest (ROI) centered on the kinetochore in the z-section where the intensity was highest. The average signal over the 25-pixel ROI was taken as the intensity measurement for that channel at a specific kinetochore. This was repeated for 10 kinetochores from distinct sister-pairs in ∼10 cells per experimental repeat. For K-fiber analysis, the same ROI was placed over the terminus of the α-tubulin signal, adjacent to the kinetochore. In all cases, an internal control, typically CENP-C, was used to normalize measurements between cells/conditions. Distance measurements were made using the “distances” tool in SoftWorx, which quantifies the distance between two manually defined 3D coordinates. Bar plots were generated in Excel (Microsoft), with error bars depicting ± SD. Box plots were generated in R, with the box depicting the median and first and third quartiles and the whiskers representing Q1 and Q3 ± 1.5× interquartile range. Intensity values are quoted as a percentage of the control ± SD. Distributions were compared using Welch’s two-sample *t* test in R. Distribution was assumed to be normal, although this was not formally tested. For Fig. 10, conditions were compared using a χ^2^ test in R.

### Live-cell imaging

To film single kinetochore-pairs, HeLa-K eGFP-CENP-A–expressing cells were seeded in FluoroDishes (World Precision) and imaged in DMEM supplemented with 10% FCS, 100 U/ml penicillin, 100 µg/ml streptomycin, and 0.1 µg/ml puromycin. 3D image stacks (25 × 0.5-µm z-sections) were acquired every 7.5 s using a 100× oil NA 1.4 objective on an Olympus Deltavision Elite microscope equipped with a eGFP and mCherry filter set, Quad-mCherry dichroic mirror, solid-state light source, and CoolSNAP HQ2 camera. Environment was tightly controlled at 37°C and 5% CO_2_ using a stage-top incubator (Tokai Hit) and a weather station (Precision Control). Image stacks were deconvolved using SoftWorx (Applied Precision Ltd.), and measurements of kinetochore velocity were taken manually from tracks of persistent movement that lasted at least three time frames. For overnight imaging, cells were seeded in FluoroDishes (World Precision) and imaged in DMEM supplemented with 10% FCS, 100 U/ml penicillin, and 100 µg/ml streptomycin. 3D image stacks (7 × 2 µm z-sections) were acquired every 3 min for 12 h on the Deltavision system described above. For the analysis of kinetochore oscillations, HeLa-K eGFP-CENP-A–expressing cells were seeded in FluoroDishes (World Precision) and imaged in L15 medium supplemented with 10% FCS, 100 U/ml penicillin, and 100 µg/ml streptomycin. 3D image stacks (25 × 0.5-µm z-sections) were acquired every 5 s for 5 min using a 100×/1.46-NA immersion oil objective on a confocal spinning disk microscope (Marianas SDC, 3i) equipped with a Photometrics 95B Prime scientific complementary metal–oxide–semiconductor camera controlled by Slidebook 6.0 (3i). Environment was tightly controlled at 37°C using a stage-top incubator and a weather station (both Okolab). Tracking and analysis of the oscillatory movements of the eGFP-CENP-A–labeled kinetochores were then performed using kinetochore tracking software KiT v2.1.15 (Olziersky et al., 2018).

### Lattice light sheet microscopy and image analysis

LAP-Spindly cells were seeded on 5-mm coverglass in 33-mm dishes, and siRNA was performed as detailed above. For the nocodazole washout, cells were arrested in 3.3 µM nocodazole for 4 h before imaging. Cells were washed for 3 min in L15 supplemented with 10% FCS, 100 U/ml penicillin, 100 µg/ml streptomycin, and 0.1 µg/ml puromycin and mounted on the microscope; imaging commenced within 5 min. Lattice light sheet microscopy was performed using a 3i Lattice LightSheet instrument. The sheet pattern was a Bessel lattice of 52 beams, with inner and outer numerical apertures of 0.493 and 0.55, respectively. 3D volumes were recorded with a 0.57-µm step size (0.308 µm deskewed) for 130 planes with a 35-ms exposure. This resulted in an overall speed of 6 s per volume. One volume was recorded every minute for 60 min. The resultant 4D image stacks were deskewed and z-projected by maximum intensity in SlideBook. Maximum-intensity projections were opened in Fiji and cropped to the cell of interest and to a single time point. TrackMate was then used as a spot detector, with a spot diameter of 0.5 µm and threshold adjusted to minimize false detections. The spot statistics output from TrackMate was saved for analysis. This process was repeated for every time point using the same threshold throughout. Intensity measurements were adjusted for bleaching with background subtraction. For visualization, all values were divided by the control *t* = 0 measurement to normalize the data to 1. Data in the text is quoted with ±SD.

### Immunoblotting

Protein extracts were prepared by liquid nitrogen grinding. Briefly, cells arrested in 330 nM nocodazole for 16 h were harvested from a 15-cm dish and resuspended in 1.5× pellet volumes of H-100 buffer (containing 50 mM Hepes, pH 7.9, 1 mM EDTA, 100 mM KCl, 10% glycerol, 1 mM MgCl_2_, and a complete protease inhibitor tablet [Roche]). Cells were ground in liquid N_2_ with a precooled mortar and pestle. The ground cell extract was collected and spun at 14,000 rpm for 30 min at 4°C, and the soluble fraction was collected. Protein concentration was determined by Bradford assay. 30 µg of extract was boiled in LDS sample buffer + reducing agent (NuPage) for 10 min and separated on a 4–12% Bis-Tris gel (NuPage) in MOPS (NuPage). Proteins were wet-transferred to a nitrocellulose membrane before blocking in 5% milk TBST (Tris-buffered saline with 0.1% Tween 20) for 1 h at room temperature. Primary antibodies were incubated overnight at 4°C in 2% milk TBST: anti-CENP-F(Ab5) (1/1,500, rabbit, Abcam), anti-CENP-F(A301-611A) (1/5,000, rabbit, Bethyl), anti-DIC (1/1,000, mouse, EMD Millipore), and anti-α-tubulin (1/10,000, mouse, Sigma-Aldrich). Membranes were washed three times in TBST before incubation with HRP-conjugated secondary antibodies for 1 h at room temperature in 2% milk TBST (1/10,000, GE Healthcare).

### Online supplemental material

Fig. S1 shows immunofluorescence microscopy images of the CENP-E rescue experiment, the K-K distance rescue experiment, and the cold-stable rescue experiment, all with CENP-F MTBD mutants. Fig. S2 shows immunofluorescence microscopy images and immunoblots of HeLa-K, CENP-F-Mut1, and CENP-FMut2 cells. Fig. S3 shows immunofluorescence microscopy images and quantification of kinetochore proximal α-tubulin intensities and CENP-C–based intersister distances in HeLa-K, CENP-F-Mut1, and CENP-F-Mut2 cells. Fig. S4 shows immunofluorescence microscopy images and quantification of kinetochore SKAP signal relative to CENP-C of HeLa-K cells treated with control or DHC siRNA. Fig. S5 shows cumulative frequency plots of congression time, metaphase duration, and nuclear envelope breakdown-anaphase in HeLa-K, CENP-F-Mut1, and CENP-F-Mut2 cells. Video 1 shows live-cell visualization of stably integrated and doxycycline-induced GFP-Spindly in siControl-treated HeLa cells. Video 2 shows live-cell visualization of stably integrated and doxycycline-induced GFP-Spindly in siCENP-F–treated HeLa cells.
